# The Structure of Psychopathology in Adolescence and Its Common Personality and Cognitive Correlates

**DOI:** 10.1037/abn0000193

**Published:** 2016-11

**Authors:** Natalie Castellanos-Ryan, Frederic N. Brière, Maeve O’Leary-Barrett, Tobias Banaschewski, Arun Bokde, Uli Bromberg, Christian Büchel, Herta Flor, Vincent Frouin, Juergen Gallinat, Hugh Garavan, Jean-Luc Martinot, Frauke Nees, Tomas Paus, Zdenka Pausova, Marcella Rietschel, Michael N. Smolka, Trevor W. Robbins, Robert Whelan, Gunter Schumann, Patricia Conrod, The IMAGEN Consortium

**Affiliations:** 1 Department of Psychoeducation, University of Montreal; 2 Department of Psychology, McGill University; 3 Department of Child and Adolescent Psychiatry and Psychotherapy, Central Institute of Mental Health, Medical Faculty Mannheim, Heidelberg University; 4 Institute of Neuroscience and Discipline of Psychiatry, School of Medicine, Trinity College; 5 Department of Systems Neuroscience, University Medical Center Hamburg-Eppendorf; 6 Department of Cognitive and Clinical Neuroscience, Central Institute of Mental Health, Medical Faculty Mannheim, Heidelberg University; 7 Neurospin, Atomic Energy and Alternative Energies Commission, Paris, France; 8 Department of Psychiatry and Psychotherapy, University Medical Center Hamburg-Eppendorf; 9 Departments of Psychiatry and Psychology, University of Vermont; 10 Imaging and Psychiatry (Unit 1000), Institut National de la Santé et de la Recherche Médicale (INSERM CEA), University Paris Sud, and Department of Adolescent Psychopathology and Medicine, Maison de Solenn, University Paris Descartes; 11 Department of Cognitive and Clinical Neuroscience, Central Institute of Mental Health, Medical Faculty Mannheim, Heidelberg University; 12 Rotman Research Institute, Departments of Psychology and Psychiatry, University of Toronto; 13 Hospital for Sick Children, University of Toronto; 14 Department of Cognitive and Clinical Neuroscience, Central Institute of Mental Health, Medical Faculty Mannheim, Heidelberg University; 15 Department of Psychiatry and the Neuroimaging Center, Technische Universität; 16 Department of Experimental Psychology, University of Cambridge; 17 Department of Psychology, University College Dublin; 18 MRC Social, Genetic, and Developmental Psychiatry (SGDP) Centre, London and Psychology & Neuroscience, King’s College London; 19 Department of Psychiatry, University of Montreal, CHU Sainte-Justine Hospital, and Department of Psychological Medicine and Psychiatry, Institute of Psychiatry, Psychology & Neuroscience, King’s College London; 20 London, United Kingdom

**Keywords:** psychopathology, comorbidity, personality, cognition, adolescence

## Abstract

The traditional view that mental disorders are distinct, categorical disorders has been challenged by evidence that disorders are highly comorbid and exist on a continuum (e.g., Caspi et al., 2014; Tackett et al., 2013). The first objective of this study was to use structural equation modeling to model the structure of psychopathology in an adolescent community-based sample (*N* = 2,144) including conduct disorder, attention-deficit/hyperactivity disorder (ADHD), oppositional-defiant disorder (ODD), obsessive–compulsive disorder, eating disorders, substance use, anxiety, depression, phobias, and other emotional symptoms, assessed at 16 years. The second objective was to identify common personality and cognitive correlates of psychopathology, assessed at 14 years. Results showed that psychopathology at 16 years fit 2 bifactor models equally well: (a) a bifactor model, reflecting a general psychopathology factor, as well as specific externalizing (representing mainly substance misuse and low ADHD) and internalizing factors; and (b) a bifactor model with a general psychopathology factor and 3 specific externalizing (representing mainly ADHD and ODD), substance use and internalizing factors. The general psychopathology factor was related to high disinhibition/impulsivity, low agreeableness, high neuroticism and hopelessness, high delay-discounting, poor response inhibition and low performance IQ. Substance use was specifically related to high novelty-seeking, sensation-seeking, extraversion, high verbal IQ, and risk-taking. Internalizing psychopathology was specifically related to high neuroticism, hopelessness and anxiety-sensitivity, low novelty-seeking and extraversion, and an attentional bias toward negatively valenced verbal stimuli. Findings reveal several nonspecific or transdiagnostic personality and cognitive factors that may be targeted in new interventions to potentially prevent the development of multiple psychopathologies.

## General Scientific Summary

This study provides support for a spectrum and common liability model of psychopathology, with findings showing that in a community sample of European adolescents: a) internalizing and externalizing psychopathology share substantial variance that can be modeled as a general psychopathology factor; and (b) that the common variance across psychopathologies is associated with personality traits related to high disinhibition/impulsivity, low agreeableness, high neuroticism and hopelessness, as well as different aspect of cognitive function, namely high delay-discounting, poor response inhibition and low performance IQ.

The traditional view that mental disorders are distinct, categorical syndromes has been challenged by evidence that many disorders are highly comorbid and exist on a continuum. Indeed, comorbidity rates are high among psychiatric disorders (Kessler, Chiu, Demler, Merikangas, & Walters, 2005) and comorbid cases represent the rule rather than the exception. This suggests that mental disorders have more in common than implied by current nosology, or at least that there may be a more parsimonious structure to psychopathology. Since the mid-90s, there has been substantial research suggesting that certain psychiatric disorders in adulthood may share substantial common variance and exist on at least two liability continuums or spectra (e.g., Kendler, Prescott, Myers, & Neale, 2003; Krueger, 1999): an externalizing dimension conveying risk for disinhibited, antisocial and/or substance use disorders, and an internalizing dimension conveying liability to experience mood and anxiety disorders. Importantly, these findings in adulthood confirmed dimensional models of psychopathology frequently used and replicated in child samples since the 1980’s (Achenbach & Edelbrock, 1981), suggesting stability of this structure across developmental periods. However, although these internalizing and externalizing dimensions have been replicated across populations and developmental periods, studies have also shown that there are significant positive correlations among these latent dimensions/factors. This has motivated a new wave of studies investigating the structure and implications of these interfactor correlations. In line with this, a number of recent studies (Carragher et al., 2016; Caspi et al., 2014; Lahey et al., 2012; Laceulle, Bollebergh, & Ormel, in press; Lahey et al., 2015; Murray, Eisner, & Ribeaud, 2016; Noordhof et al., 2015; Patalay et al., 2015; Pettersson, Lahey, Larsson, Lundström, & Lichtenstein, 2015; Tackett et al., 2013; Wright & Simms, 2015), have shown that psychopathology diagnoses and symptoms may be best modeled with a bifactor (or general-specific) model, which includes (a) a general factor capturing the common variance shared across all symptoms, and (b) specific factors reflecting the additional shared variance among symptoms, be they internalizing and externalizing factors (Lahey et al., 2015; Patalay et al., 2015; Tackett et al., 2013), externalizing, internalizing and thought disorder factors (Carragher et al., 2016; Caspi et al., 2014; Laceulle et al., in press), externalizing, distress and fear factors (Lahey et al., 2012), or other factors (Murray et al., 2016; Noordhof et al., 2015; Wright & Simms, 2015). The replication of findings across studies is noteworthy given the range of samples and psychopathology disorders/symptoms included. Remarkably, these studies demonstrated that a general psychopathology factor (or P factor, as coined by Caspi et al., 2014) can be reliably replicated across samples and psychopathology disorders/symptoms, as well as developmental periods. Indeed, Murray, Eisner, and Ribeaud (2016) recently demonstrated that the P factor remained stable from age 7 to 16 years. Finally, these recent studies have also provided initial insights into the nature of the shared variance across psychopathology. For example, higher scores on the P factor were associated with greater life impairment (Caspi et al., 2014), lower school attainment, and higher psychopathology later in life (Patalay et al., 2015; Pettersson et al., 2015), greater economic deprivation in childhood (Caspi et al., 2014; Patalay et al., 2015) and lower IQ and school functioning (Caspi et al., 2014; Lahey et al., 2015), as well as higher neuroticism and lower agreeableness and conscientiousness (Caspi et al., 2014). Using a behavioral genetics design, Tackett et al. (2013) found that high scores on the P factor were associated with higher negative emotionality, and that there was substantial variance shared between the P factor and negative emotionality at both phenotypic and genetic levels.

Taken together, these findings suggest that a general psychopathology factor can be modeled across different measures of psychopathology, is robust across developmental periods, reflects shared personality and IQ correlates, as well as environmental and genetic influences, and may improve prediction of future individual functioning. However, the nature of a general psychopathology factor in adolescence remains understudied. Importantly, although there are a number of studies that include adolescent samples (Carragher et al., 2016; Laceulle et al., in press; Murray et al., 2016; Noordhof et al., 2015; Patalay et al., 2015; Tackett et al., 2013), only one study so far has modeled substance use (i.e., alcohol use problems, Carragher et al., 2016), and none has included eating disorders. This is an important limitation since the peak of onset for many psychopathology disorders, particularly the onset of substance use (SU) problems (Kessler, Berglund, et al., 2005) and eating disorders (Nagl et al., 2016; Stice et al., 2009) occurs during adolescence.

Second, only two studies have examined the personality correlates of a general psychopathology factor in adolescence. As previously mentioned, Tackett et al. (2013) found that negative emotionality may be associated with a liability to developing a range of psychopathologies, which is in line with the wider personality-psychopathology literature showing that neuroticism is the Big Five trait most strongly associated with many forms of psychopathology, particularly mood and anxiety disorders (Widiger & Costa, 1994; Kotov, Gamez, Schmidt, & Watson, 2010), as well as their comorbidity (Khan, Jaconson, Gradner, Prescott, & Kendler, 2005). Low conscientiousness and low extraversion have also consistently been associated with several mental health disorders (Malouff, Thorsteinsson, & Schutte, 2005; Kotov et al., 2010). Carragher et al. (2016) also modeled the common variance across several symptoms of emotional, hyperactive, and conduct problems in Australian youth and found that several lower-order personality traits were associated with the common variance across these symptoms, with hopelessness and impulsivity being the strongest predictors. This finding is consistent with other studies showing associations between these lower-order factors and a range of psychopathologies (Castellanos-Ryan, O’Leary-Barrett, Sully, & Conrod, 2013; Kuo, Gallo, & Eaton, 2004) and their comorbidity (Castellanos-Ryan & Conrod, 2012; Castellanos-Ryan et al., 2014). A limitation of these two studies, however, is that assessments have been concurrent, which precludes drawing conclusions regarding the temporality of the associations between personality traits and the P factor. Other limitations are that these studies have not included both higher- and lower-order personality dimensions in relation to general-specific psychopathology factors and have not included a wide range of potential substance use problems, although these problems appear to be related to a distinct personality profile compared to many other forms of psychopathology—in that they are less related to neuroticism but more elevated on disinhibition, sensation-seeking, and disagreeableness (Castellanos-Ryan & Conrod, 2012; Kotov et al., 2010). Thus, a longitudinal examination of associations between general and specific dimensions of psychopathology, which include a wider range of disorders and personality measures, would be useful.

Third, very few studies have been designed and sufficiently powered to examine the cognitive correlates of psychopathology when modeled hierarchically. Due to its large scale and comprehensive cognitive assessment battery, the IMAGEN study provides a unique opportunity to examine the hierarchical structure of psychopathology and the cognitive correlates of such latent dimensions. We previously reported on the cognitive and functional imaging correlates of the latent structure of externalizing problems using this large sample of European adolescents (Castellanos-Ryan et al., 2014). A general-specific factor was supported by structural equation modeling, and neuropsychological and functional imaging tasks were shown to dissociate the three latent factors concurrently and longitudinally. A general externalizing factor was specifically associated with delay discounting and hyperactivation of the presupplementary motor cortex (coupled with hypoactivity of the substantial nigra and subthalamic nucleus) during successful behavioral inhibition. However, because this study only focused on externalizing symptoms, it is unclear to what extent this externalizing factor captured shared variance between externalizing symptoms that is in fact shared by a wider range of psychopathology symptoms and could be captured by a more general psychopathology factor. It also did not allow the identification of the cognitive correlates that are associated to the shared variance across externalizing and internalizing symptoms. Caspi et al. (2014) examined the associations between the P factor and cognitive functions and other brain measures (i.e., retinal imaging), and showed that a derived P factor correlated negatively with adult and child measures of cognitive function and brain integrity generally. Similarly, Lahey et al. (2015) found that the P factor modeled in childhood was also associated with poor verbal and spatial IQ. These results are consistent with studies on individual disorders showing that lower intelligence and cognitive impairments, such as deficits in response inhibition and working memory, are shared by a wide range of externalizing problems, such as aggression and ADHD/CD (Lijffijt et al., 2005; Oosterlaan & Sergeant, 1998; Peeters, Monshouwer, Janssen, Wiers, & Vollebergh, 2014; Young et al., 2009) and internalizing disorders, such as depression (Snyder, 2013; Wagner et al., 2015) and various anxiety disorders including social anxiety disorder, generalized anxiety disorder, obsessive–compulsive disorder, and posttraumatic stress disorder (Ferreri, Lapp, & Peretti, 2011; Olley, Malhi, & Sachdev, 2007; Polak, Witteveen, Reitsma, & Olff, 2012). However, other cognitive risk factors which have been investigated in relation to individual disorders may be more specific to internalizing or externalizing dimensional factors. For instance, attentional biases toward stimuli with a negative emotional valence have been associated with several internalizing problems, but may be less characteristic of externalizing problems (Peckham, McHugh, & Otto, 2010). On the other hand, deficits related to delay discounting or risky decision making may be more specific to externalizing problems (Bickel, Koffarnus, Moody, & Wilson, 2014; Castellanos-Ryan et al., 2014). However, these cognitive variables have never been examined in previous bifactor studies. Interestingly, Caspi, et al. (2014) found no correlation between a specific externalizing factor and any brain measures, which is unexpected considering the large literature on cognitive and brain abnormalities in ADHD and CD (e.g., Castellanos-Ryan, Rubia, & Conrod, 2011) and the findings reported by Castellanos-Ryan et al. (2014) suggesting that significant cognitive impairment, particularly poor response inhibition, is mediated by hypofunction of prefrontal cortical circuits. However, this could be due to the fact that specific externalizing behavior in the Caspi et al. (2014) study was assessed mostly with substance use behaviors and included only conduct problems, but not ADHD, as a nonsubstance use-related externalizing problem. Additional structural studies which include a wider range of potential externalizing symptoms are required to clarify this question.

Within this context, our first aim was to model the structure of dimensional psychopathology in adolescence and determine whether it is best described by a general-specific bifactor model, as found in previous studies. As adolescence is a developmental period characterized by important biological, cognitive, emotional and social changes, and the peak of onset for many psychopathology disorders, particularly the onset of SU problems (Kessler, Berglund, et al., 2005) and eating disorders (Nagl et al., 2016; Stice et al., 2009), we also aimed to examine the stability of the model across the transitional period of early- to mid-adolescence. To do this, we model the structure of psychopathology at two different ages in adolescence (14 and 16 years) in a community-based European sample including a diverse range of mental disorder symptoms. Importantly, we examine how three different substance misuse indicators (alcohol problems, drug use and tobacco use) and eating disorders integrate into a bifactor psychopathology model in adolescence, which has not been done before. Our third aim is to examine the prospective association between cognitive and personality correlates at age 14 and general-specific factors at age 16. Due to the extensive neuropsychological battery used in the IMAGEN study, the current analysis is unique in its ability to assess the extent to which delay discounting, risky decision making and attentional biases to positive and negative emotional stimuli (which have previously been associated with neuroticism) are associated with the general P factor or more specific factors. In this way, we will investigate the relationships between specific adolescent liability factors and cognition, beyond IQ and executive function, which has never been done before. We will also examine the prospective link between higher- and lower-order personality measures and general-specific psychopathology factors, which has not been done in previous bifactor studies. A final contribution of this study is to put our previous findings (Castellanos-Ryan et al., 2014) into context with respect to the personality and cognitive correlates of externalizing psychopathology, when models of psychopathology consider the internalizing spectrum in addition to the externalizing spectrum and their common variance.

These objectives will hopefully lead to a better understanding of common and specific correlates of psychopathology that can inform the development of new interventions (as we have done successfully for externalizing neurophenotypes; e.g., Conrod et al., 2013; O’Leary-Barrett et al., 2013) that could potentially impact a multitude of outcomes by targeting personality and cognitive risk dimensions.

## Method

### Participants

A total of 2,232 participants aged 14 years across eight European sites were recruited via high-schools. Parents gave informed written consent and adolescents gave written assent to the study procedure prior to inclusion. All procedures were approved by each local institutional ethics committee. Further descriptions of the study design, sample, and recruitment procedure, including data storage and safety can be found in the Supplementary Material and elsewhere (Schumann et al., 2010).

After data quality control, complete and reliable data sets for 2,144 volunteers with an average age of 14.39 years (*SD* = 0.77) and an even sex ratio (*n* = 1,093 girls, i.e., 51%) were included in analyses. Reliable follow-up data was gathered for 1,603 (75%) participants at 16 years. Participants who were followed up did not differ significantly from those not followed on demographic, psychopathology symptoms, or cognitive variables, except for language (Odds Ratio = 2.79, 95% CI [2.20, 3.55], with English speakers being more likely to be followed) and parent-reported ADHD symptoms (Odds Ratio = 0.93, 95% CI [0.87, 0.99], with those scoring higher being less likely to be followed). All participants for whom we had reliable data at 14 years (*N* = 2,144) were included in analyses.

### Measures

All measures were selected on the basis of brevity, age-appropriateness, and validity in their variant forms (English, German, and French). Psychopathology and substance misuse symptoms over the last 12 months were assessed at 14 and 16 years of age. Personality and cognitive function were only assessed at 14 years.

#### Psychopathology symptoms

Self- and parent-report behavioral and clinical measures were assessed via online computer platforms provided by Psytools ® (Delosis Ltd, London, United Kingdom), administered at participants’ homes, and The Development and Well-Being Assessment interview (DAWBA, Goodman, Heiervang, Collishaw, & Goodman, 2011; see also http://www.dawba.info), was administered at the research site. The DAWBA interview was administered to adolescents and parents and assessed psychiatric symptoms of CD, ODD, ADHD, generalized anxiety, depression, specific phobia, social phobia, agoraphobia, panic disorder, OCD, and eating disorders. A prognosis for the likelihood of having a disorder was calculated by computer algorithms that use the symptoms and impact recorded in the structured sections of both the parent-rated and self-rated DAWBA. These computer-generated band scores range from level 0 up to level 5, corresponding to the approximate prevalence rates in an epidemiological sample for the disorder in question (ranging from less than 0.1% up to 70%). Diagnostic criteria were based on the *Diagnostic Statistical Manual*, Version 4. Because of low prevalence, likelihood of specific phobia, agoraphobia and panic disorder were averaged to create a combined “panic and other phobia” score. This was deemed justified as these three band scores were associated similarly to all other psychopathology indicators and correlates in this sample.

#### Substance misuse

Substance misuse was assessed using the Alcohol Use Disorders Identification Test (AUDIT; Saunders, Aasland, Babor, de la Fuente, & Grant, 1993) and the European School Survey Project on Alcohol and Drugs (ESPAD; Hibell et al., 1997). The AUDIT was developed and validated by the World Health Organization to provide a brief assessment of alcohol use disorders and was specifically designed for international use. It exists in all three languages, and has been validated on primary health care patients and community samples. For this study, the scale total for problematic or harmful alcohol use in the last year included feelings of guilt or remorse after drinking, being unable to remember what happened the night before because of drinking, being injured or having injured someone as a result of drinking and relevant others being concerned about their drinking and suggestions to cut down. The ESPAD items used in this study comprised tobacco use frequency and the number of drugs used over the last 12 months (see Table 1 for prevalence and correlations between all psychopathology measures at 16 years).[Table-anchor tbl1]

#### Personality

Personality was assessed with the self-reported Substance Use Risk Profile Scale (SURPS; Woicik, Stewart, Pihl, & Conrod, 2009), the NEO Five Factor Inventory (NEO-FFI; Costa Jr & McCrae, 1992), and the Temperament and Character Inventory (TCI; Cloninger, Przybeck, Svrakic, & Wetzel, 1994). The SURPS assessed the personality traits of hopelessness, anxiety sensitivity, impulsivity, and sensation-seeking. The reliability and concurrent and predictive validity of this measure has been well established in several adolescent and adult samples in different countries (Castellanos-Ryan et al., 2013; Krank et al., 2011; Woicik et al., 2009). The NEO-FFI assessed five higher order personality characteristics: neuroticism, conscientiousness, extraversion, agreeableness, and openness to experience. The TCI was used to assess novelty-seeking, which is considered a good general measure of impulsive tendencies that also includes sensation-seeking (see Table S2 in supplementary material for correlations between personality and cognitive measures).

#### IQ and cognitive measures

Estimates of *intelligence* were derived from the vocabulary and similarities subtests (verbal IQ) and block design and matrix reasoning subtests (performance IQ) of the Wechsler Intelligence Scale for Children—4th edition (WISC-IV; Wechsler, 2003). Digit span forward and backward subtests from the WISC-IV were also administered and used to assess short-term auditory memory and auditory working memory (Groth-Marnat & Baker, 2003; Reynolds, 1997). *Poor response inhibition* was measured using the number of commission errors in a go/no-go passive avoidance learning paradigm (Newman & Wallace, 1993). *Delay discounting* was assessed with the Kirby Delay Discounting Questionnaire (Kirby, Petry, & Bickel, 1999). This measure was scored as described previously by Kirby, Petry, and Bickel (1999), with k values (an index of delay discounting) assigned according to choice patterns across the 27 items.

*Spatial working memory, risky decision-making, and information processing biases for positive and negative stimuli* were assessed with three tasks from the Cambridge Cognition Neuropsychological Test Automated Battery (CANTAB; Cambridge Cognition), the Spatial Working Memory (SWM), Cambridge Gambling task, and affective go/no-go task, respectively. SWM is a self-ordered test that requires retention and manipulation of visuospatial information. A modified version of the Cambridge Gambling Task (which reduced the time between stakes from 5 s to 2 s to make the task shorter to avoid boredom effects in adolescents) was used to assess risky decision-making. Finally, the affective go/no-go is a task of emotional processing, in which affectively valenced words (happy and sad) are presented one at a time on screen. Performance variables-of-interest are the target (omission) errors to positive and negative words, with an attentional bias toward negative versus positive words being assessed with a difference score between omission errors to each set of stimuli. For further details on the cognitive tasks see Supplementary Material and http://www.cambridgecognition.com/academic/cantabsuite.

### Data Analysis

A series of structural equation models on computer-generated scaled likelihood of diagnosis scores and self-reported substance use were analyzed using MPlus version 7.11 (Muthén & Muthén, 2010). Based on previously reported theoretical models and analyses (e.g., Carragher et al., 2016; Caspi et al., 2014; Castellanos-Ryan et al., 2014; Lahey et al., 2012; Lahey et al., 2015; Tackett et al., 2013), several models were assessed for goodness of fit: (a) a single “psychopathology” factor loading on all indicators; (b) two correlated factor models, where variables assessing internalizing (INT) psychopathology (generalized anxiety, depression, social phobia, panic and other phobias, OCD and eating disorders)^1^ and CD, ODD, ADHD, and substance use (SU) loaded on two specific INT and EXT factors (with SU variables loading on the EXT factor; Model 2a) or loaded on three specific INT, externalizing (EXT) and SU factors (Model 2b). Model 2a and 2b allowed subfactors to covary. Lastly (c), two bifactor (or general-specific) models were assessed, in which a general psychopathology factor (P) was added at the same level as the specific factors from the previous (Models 2a and 2b) models (Models 3a and 3b). In these last models, factors were not allowed to covary (i.e., they were constrained to zero), consistent with a classic bifactor model, but because many recent studies present modified versions of bifactor models (e.g., Carragher et al., 2016; Caspi et al., 2014), in which the specific factors are allowed to covary, two final revised bifactors models (Models 3a′ and 3b′) that allowed the specific factors to correlate were also examined (see Figures S1–S7 in supplementary material for a graphic representation of all models tested). In all models, gender and language (English vs. other) were entered as covariates (at the level of observed variables). In addition, all models were fit using a complex random effects design to control for testing site as a cluster variable, and used robust maximum likelihood estimation (MLR). MLR has been shown to perform well when modeling low prevalent behaviors or nonnormal data (Asparouhov & Muthén, 2005). Full information maximum likelihood was used to handle missing data.

Once the best fitting model was established, two sets of correlates (personality and cognitive indices) were each entered into the model separately. That is, unadjusted associations were examined by entering the personality and cognitive variables and the psychopathology factors into the same model and allowing them to correlate. Adjusted associations were examined in four separate models in which the psychopathology factors were regressed onto (a) all SURPS subscales; (b) all NEO subscales; (c) novelty seeking (on its own); and (d) all cognitive variables entered together in the same model. The Benjamini-Hochberg procedure (Benjamini & Hochberg, 1995) was used to correct for multiple testing. Once *p* values are sorted in ascending values, the Benjamini-Hochberg procedure allows one to calculate the false discovery rate (FDR) for each of the *p* values (i.e., at each “position” in the sorted list of *p* values, it will indicate what proportion of those are likely to be false rejections of the null hypothesis). This procedure to control for multiple testing has been shown to be less stringent and have more power than Bonferroni correction or other types of familywise error rate corrections (see online Supplementary Material for further description of the sample, measures and analytic approach).

## Results

### The Structure of Psychopathology in Adolescence

**Figure 1 fig1:**
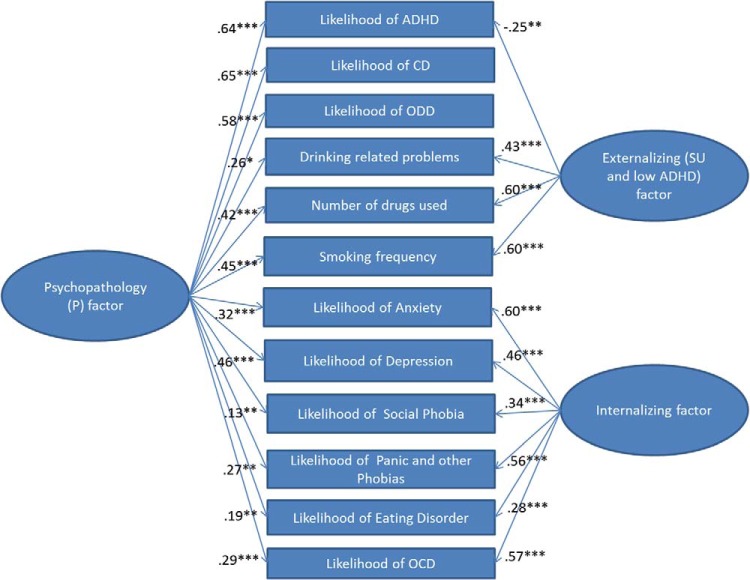
Bifactor Model 3a of psychopathology. ADHD = attention-deficit/hyperactivity disorder; CD = conduct disorder; ODD = oppositional defiant disorder; OCD = obsessive compulsive disorder; SU = substance use. * *p* < .05. ** *p* < .01. *** *p* < .001. See the online article for the color version of this figure.

Of the models tested, the three correlated factor model (Model 2b) and all bifactor models (both classic or modified) fit the data well, with the bifactor model with the EXT and INT specific factors (Models 3a and 3a′) or the modified bifactor model with the three specific ADHD/CD/ODD, SU, and INT factors fitting the data best (see Table 2). The fit was equally good for these three models, but Model 3a (see Figure 1) was chosen over Model 3a′ because of parsimony and the fact that the additional correlation did not improve model fit and was nonsignificant). Factor loadings for Model 3a appear in Table 3, and factor loadings for Model 3b′ appear in Table S3 in supplementary material. In both models, all psychopathology indicators loaded significantly on the P factor, with CD, ODD, ADHD, smoking frequency, numbers of drugs used, and depression loading the strongest on this factor (≥.42), and eating disorders and social phobia loading the weakest on this factor (≤.22). Indeed, at 16 years, the P factor represented mainly the substantial common variance shared among externalizing disorders (including smoking and drug use), depression and, to a slightly lesser extent, anxiety, OCD, phobias, and drinking problems. The specific INT factors in both Models 3a and 3b′ reflected variance unique to all internalizing problem (albeit a low loading for eating disorders) measures. In Model 3a the specific EXT factor reflected variance unique to all substance use measures and a low likelihood of ADHD diagnosis, whereas in Model 3b′, the specific SU and EXT factors respectively reflected the variance unique to all substance use measures and or variance unique to ADHD, ODD, and CD. In Model 3b′, which allowed specific factors to covary, the specific SU factor was negatively associated with the specific INT factor (*r* = −.27, *p* = .014) and, the specific EXT factor at a trend level (*r* = −.15, *p* = .085); and the specific EXT and INT factors were not significantly associated (*r* = .26, *p* = .554). The negative loading of ADHD and ODD on the specific EXT factor in Model 3a and the negative correlation between the specific EXT and SU factors in Model 3b′ suggest that, once the common variance shared among disorders is removed by the P factor, SU is associated with a lower likelihood of having ADHD and ODD. This is not the case for CD though, with modification indices for both models suggesting that there is some small residual variance shared (positive correlations) between CD and all SU items. Both models fit the data well and have advantages and disadvantages: Model 3a is more parsimonious but includes positive and negative loadings on the EXT factor, while Model 3b′ includes factors with only positive loadings and is easier to interpret, but very little variance is captured by the specific EXT factor, which does not differ significantly from zero (*p* = .694). Because of parsimony, and the fact that all of its factors had good variability, results for Model 3a are provided in the main text, while results for Model 3b′ are presented as Supplementary Material.[Table-anchor tbl2][Fig-anchor fig1][Table-anchor tbl3]

Taken together, results for both models suggest that most of the variance common to all externalizing symptoms is accounted by a general psychopathology (P) factor rather than an externalizing factor in this sample of adolescents. Once the P factor was modeled, the externalizing factor captured variance specific to substance misuse symptoms (in Model 3a), rather than the variance shared across all externalizing disorders. Thus, although referred to as a specific externalizing (EXT) factor, this factor in Model 3a really represents a substance use and low ADHD factor.

### How Stable is This Structure From Early (14 Years) to Middle (16 Years) Adolescence?

At 14 years the two specific factor bifactor model of psychopathology (Model 3a) also fit the data well, χ^2^(42) = 53.30, CFI = 1.00, RMSEA = .011; SRMR = .016; BIC = 61037.87; Adj BIC = 60793.22, resulting in very similar loadings to those found at 16 years, with just slightly lower loadings for most internalizing symptoms on the P factor (see table S4 in supplementary material). Correlations between factors at 14 years and 16 years showed that factors were largely stable over 2 years, with nonsignificant or only small longitudinal correlations across factors (see bottom of Table 3). However, although largely stable across time, these factors were not found to be metrically invariant over time. That is, when factor loadings were constrained to be equal across time (i.e., weak factorial invariance) the model fit was significantly worsened relative to when they were freely estimated (χ^2^diff = 146.68, DFdiff = 24, *p* < .001). Thus, after an inspection of the factor loading at 14 and 16 years, the loadings that did not demonstrate configural invariance (i.e., that differed across time) were freed, to test whether partial factorial invariance could be met. The model in which loadings for all internalizing indicators, except for eating disorders, number of drugs used and tobacco use were allowed to be freely estimated over time did not differ significantly from the base, freely estimated model (χ^2^diff = 17.13, DFdiff = 10, *p* = .072), indicating that the loadings for ADHD, CD, ODD, drinking problems, and eating disorder did not differ across time. Taken together, these results suggest that while scores within factors were stable across time and very little longitudinal association existed across factors, and the P factor bifactor structure fits well at both 14 and 16 years, the size of the contribution of psychopathology symptoms or indicators to the P factor may vary across development, with internalizing symptoms and drug and tobacco use becoming stronger with increasing age.

### Unadjusted Personality and Cognitive Correlates of Psychopathology

All predictor models with covariates showed good model fit (see note under Table 4). Table 4 presents correlations between covariates and the bifactor model of psychopathology from Model 3a (associations with factors from Model 3B’ can be found in Table S5 in supplementary material). Results showed that after controlling for multiple testing, common variance across psychopathology (P factor) was significantly associated with high levels of impulsivity, novelty-seeking, neuroticism, hopelessness, sensation-seeking, and extraversion, and low levels of agreeableness and conscientiousness. The P factor was also associated with high delay-discounting, low verbal and performance IQ, low working memory (spatial and verbal), poor response inhibition and risk-taking. Unique variance for EXT (SU and low ADHD) symptoms was significantly associated with high novelty-seeking, sensation-seeking, and extraversion, high verbal IQ and high risk-taking. In contrast, unique variance for INT symptoms was associated with high neuroticism, hopelessness and anxiety sensitivity, low novelty-seeking and extraversion, high conscientiousness, high attention (as measured by digit-span forward), and an attentional bias toward negatively valenced verbal stimuli.[Table-anchor tbl4]

### Adjusted Associations Between Personality, Cognitive Correlates, and Psychopathology

In order to examine whether associations between personality, cognitive correlates, and psychopathology factors remained once the effects of other personality and cognitive characteristics were adjusted for, different path analyses were conducted where correlations between correlates and factors were substituted by regression paths in the models. That is, for example, one model was conducted where all cognitive characteristics were entered as predictors and the psychopathology factor being regressed on all cognitive correlates simultaneously. Because of high correlations between some personality traits across measures (e.g., *r* = .47 between neuroticism and hopelessness, see supplementary table S2) and for ease of comparability with previous findings, separate models were conducted in which psychopathology factors were regressed on personality traits from each personality measure. Adjusted associations between correlates at 14 years and psychopathology factors at 16 years (see the second part of Table 4), showed that the P factor was predicted by high impulsivity, novelty-seeking, extraversion, hopelessness, and neuroticism, and low agreeableness, conscientiousness, and anxiety sensitivity. The P factor was also predicted by low spatial IQ, high delay discounting, and poor response inhibition. The specific EXT (SU and low ADHD) factor was predicted by high novelty-seeking, sensation seeking, and extraversion, and high verbal IQ and high risk-taking (gambling task). Finally, the specific INT factor was associated with high neuroticism, hopelessness, conscientiousness, anxiety sensitivity, and low novelty-seeking and extraversion. The INT factor was also associated with an attentional bias toward negatively valenced verbal stimuli. In these models, personality traits explained 8% to 15% of the variance of the P factor, 3% to 7% of the variance of the EXT (SU low ADHD) factor and 4% to 14% of the variance of the INT factor. Cognitive correlates explained 6%, 2%, and 5% of the variance of the P factor, EXT (SU and low ADHD) factor, and INT factor, respectively.

## Discussion

The first objectives of the current study were to model the structure of psychopathology in a community sample of European adolescents, and to examine the stability of psychopathology symptoms from early to middle adolescence (14 to 16 years). Findings demonstrated that a general psychopathology (P) factor can be modeled in this community adolescent sample, as well as either (a) two specific externalizing and internalizing factors or (b) three specific ADHD/CD/ODD, substance use, and internalizing factors, providing further support for a spectrum and latent trait model of psychopathology (e.g., Caspi et al., 2014; Lahey et al., 2012, 2015; Murray et al., 2016). This study contributed to the literature by extending previous bifactor models to include eating disorders and a wider range of substance use symptoms. This study also showed that a bifactor structure of psychopathology was stable across early-to-middle adolescence, a period characterized by substantial change and the onset of many disorders, but that the contributions made by different psychopathology symptoms to the P factor changed across development. Indeed, longitudinal factorial invariance analyses conducted in the present sample showed that loadings for internalizing symptoms, as well as drug use and tobacco use, became stronger with age.

Of note, although like other studies we found that a bifactor model of psychopathology, with either two or three specific factors fit the data well, our findings differ from previously reported P factor models in the following ways: (a) externalizing symptoms loaded more strongly on the P factor in this study, rather than internalizing symptom, which has been the case for many studies modeling the P factor (e.g., Caspi et al., 2014; Lahey et al., 2012); and (b) when only two specific EXT and INT factors were modeled, the EXT factor included significant positive loadings for SU variables but negative loadings for ADHD and ODD. These discrepancies could reflect differences across sample demographics, measures used, and/or developmental stage. Future studies on this sample could examine the structure of psychopathology using different indicators (e.g., symptom scores) and test for factorial invariance across countries to aid in confirming these hypotheses. That said, this study joins the fast growing literature confirming that, regardless of symptoms/disorders measured, there is substantial variance shared among these that can be captured by a general psychopathology factor.

Interestingly, the P factor in this study accounted for the common variance across externalizing symptoms that was previously attributed to a general externalizing factor in another IMAGEN study focusing specifically on the structure of externalizing symptoms (Castellanos-Ryan et al., 2014). This finding highlights that modeling the structure of psychopathology based on a broad range of symptoms may clarify the nature, antecedents, and implications of liabilities to multiple psychiatric problems, which may have been incompletely captured by narrower analyses (e.g., modeling just the EXT or INT spectrums). Our results suggest that nonsubstance-related externalizing problems (i.e., CD, ADHD, and ODD) may not have more in common with each other and with substance use problems than the general liability to psychopathology shared with internalizing problems.

Another objective was to identify some of the common and unique personality and cognitive correlates of general and specific psychopathology factors. Findings showed that after controlling for multiple testing, common variance across psychopathology was generally related to most personality measures, with the exception of openness to experience and anxiety sensitivity, in theoretically expected ways and in line with previous findings. Namely, the P factor was associated positively with neuroticism, hopelessness, impulsivity, novelty-seeking, and negatively with agreeableness and conscientiousness (Carragher et al., 2016; Caspi et al., 2014; Tackett et al., 2013). These associations remained largely unchanged after adjusting for other personality traits in the model, suggesting that the general liability to psychopathology may be characterized by a dysregulated personality profile involving high negative affect, low positive affect and poor behavioral control.

In terms of cognitive correlates, unadjusted findings also replicate the pattern of results suggesting that the P factor was associated with poor general cognitive functioning (Caspi et al., 2014). Adjusted associations showed that high-delay discounting, poor response inhibition, and low performance IQ were uniquely associated with the general psychopathology factor in this sample of adolescents. These findings are consistent with those of Caspi et al. (2014) and Lahey et al. (2015) identifying low performance IQ and poor executive function as important correlates of a general psychopathology factor. Delay discounting has not previously been examined as a correlate of a general liability to psychopathology within a spectrum or bifactor methodology framework, but this finding echoes studies identifying poor delay discounting as an important nonspecific risk factor for psychopathology (or transdisease mechanism; e.g., Bickel, Koffarnus, Moody, & Wilson, 2014; Castellanos-Ryan et al., 2014).

Correlates of specific factors were also identified, with high sensation-seeking, high verbal IQ, and risk-taking being related to variance specific to substance misuse, and high neuroticism, hopelessness, anxiety-sensitivity, conscientiousness, and agreeableness but low novelty seeking and extraversion, as well as an attentional bias toward negatively valenced verbal stimuli being associated with variance specific to internalizing disorders. These findings of dissociation in the cognitive profiles of specific substance use factors from general externalizing, or in this case psychopathology factors, are consistent with previous analyses of this and other adolescent samples (Castellanos-Ryan & Conrod, 2012; Castellanos-Ryan et al., 2014) showing that sensation-seeking and individual differences in reward responding and impulsive choice (as assessed by risk-taking on a gambling task) specifically predict vulnerability to substance use in adolescence, whereas measures of response inhibition or “impulsive action” predict variance in other externalizing problems and/or their co-occurrence. The result that neuroticism, hopelessness, anxiety-sensitivity, and low extraversion are generally associated with internalizing symptoms is consistent with previous studies and reviews (Khan et al., 2005; Kotov et al., 2010; Malouff, Thorsteinsson, & Schutte, 2005; Naragon-Gainey, 2010). This is the first study that we know of to identify a cognitive correlate for the common variance across internalizing disorders in a bifactor framework. Attentional biases toward negative stimuli have been consistently associated with depression (e.g., Joormann & Quinn, 2014), but our study is the first to show that such biases represent a transdiagnostic risk factor for depression, multiple types of anxious symptoms, and eating disorder symptoms—but not externalizing problems. Although this effect has been proposed in many heuristic models of psychopathology, it has been difficult to demonstrate until very recently, likely due to the lack of studies with sufficiently large and richly characterized samples.

The strengths of this study include the large sample size within a homogeneous population of 14-year-olds assessed prospectively using a well-validated structured psychiatric assessment involving child and parent reports. This richly phenotyped sample also provides a unique opportunity to investigate the phenotypic structure of a variety of different forms of psychopathology and the cognitive correlates within this model. However, there are also a number of limitations to this population-based approach. First, although the sample is ethnically homogeneous (Caucasian), and thus findings could generalize only to a Caucasian adolescent population, there is heterogeneity in that adolescents are recruited from different cities in Germany, France, Ireland, and United Kingdom. In order to control for differences in these subsamples of adolescents, complex random effects analyses controlling for testing site as a cluster variable were conducted. However, it is important to note that findings on this combined sample may not relate to a specific population of reference in the usual sense. Future studies should test the factorial and other invariance across countries to determine how representative these findings are for each of the subsamples of adolescents. Second, although a wide range of psychopathology symptoms and measures were included in the current paper (including eating disorders that had yet to be tested), the current study did not have any validated measures of psychosis, mania, or schizophrenia (included in some shape or form in Carragher et al., 2016; Caspi et al., 2014; Forbush & Watson, 2013; Laceulle et al., in press; Wright & Simms, 2015). Thus, while we call this dimension the P factor, to be consistent with other literature (e.g., Caspi et al., 2014), we also caution that without inclusion of information on thought disorder, the P factor presented in our final model is incomplete, and for the moment might be better referred to as a general behavioral/emotional dysregulation dimension. Additional follow-ups would help us to understand how these latent factors transform as adolescents transition to adulthood and may begin to experience psychiatric problems more typically seen in adulthood. Third, our analytic strategy did not allow us to model more specific subfactors of internalizing problems (e.g., the distinction between “fear” and “distress;” Krueger, 1999). Future studies should investigate the relevance of these subfactors in a complete bifactor framework, as we have done for externalizing problems (e.g., Castellanos-Ryan et al., 2014). In addition, we note that in the current analyses we did not include exactly the same externalizing indicators as those included in our previously reported externalizing factor structure on this same sample (Castellanos-Ryan et al., 2014), resulting in somewhat different findings (i.e., in the current analyses the model including a specific ADHD/CD/ODD factor fit well, but did not capture a significant amount of variance). In our previous analysis, more variable self and parent reported “screen” ratings of ADHD and CD symptoms, as assessed by the Strengths and Difficulties Questionnaire, were also included in the analysis, which were the indicators that loaded the strongest on a specific ADHD/CD factor. It appears that when more variable screen data from the whole population are used (rather than diagnoses based on a screened sample), a general externalizing dimension can be meaningfully distinguished from an ADHD/CD-specific factor, including on cognitive and neural correlates. When focusing analyses on data representing likelihood of psychiatric diagnosis, this general externalizing factor is lost, and instead a general psychopathology factor is identified, on which ADHD and CD diagnoses dominate, but concurrent substance use and internalizing psychopathology is also captured.

## Conclusions and Clinical Implications

The findings on the personality and cognitive correlates of the P factor (a) provide additional support for the criterion validity of a general psychopathology factor, and demonstrate that the general psychopathology factor is not a spurious artifact of measurement error; and (b) offer important clues as to the psychobiological processes that may underlie the general and specific factors of psychopathology. Indeed, the current study identifies common personality and cognitive correlates underlying different dimensions of psychopathology which can inform research on the etiology of mental disorders and eventually inform the development of new intervention strategies that better address comorbidity in clinical practice by targeting transdiagnostic risk factors. The findings reported herein suggest that traits related to disinhibition/impulsivity, low agreeableness, and high negative affect, as well as high delay discounting and poor response inhibition, might underlie common vulnerability to psychopathology. Finally, targets for specific patterns of psychopathology were confirmed or newly identified and overlap with some of the dimensions identified within the research domain criteria framework (Insel et al., 2010). Specifically, targeting sensation seeking/reward sensitivity/impulsive choice appears a relevant target for specific risk for substance misuse, and managing anxiety sensitivity/attentional biases to negative stimuli might be helpful in reducing risk for internalizing psychopathology. These conclusions perfectly align with new findings on trait-targeted interventions reported in randomized controlled trials (Conrod et al., 2013; O’Leary-Barrett et al., 2013; Olthuis, Watt, Mackinnon, & Stewart, 2014, 2015). These authors have all shown that cognitive–behavioral interventions targeting these personality and cognitive profiles are effective in treating or preventing externalizing and internalizing psychopathology and substance misuse. It is worth noting that the trait-focused interventions across these studies all involved very different treatment delivery methods, including face-to-face and distance delivered (e.g., telephone) individual interventions, and school-based group interventions. This approach might also prove effective and efficient in reducing concurrent problems in general mental health clinics where clients often present with a variety of mental health concerns, and may not meet full diagnostic criteria. The dimensional approach to treatment presents advantages for these cases, where interventions can be offered rapidly, based on brief personality, cognitive or psychiatric screens, and might provide more clarity on primary source of impairment among many concurrent diagnoses. Furthermore, the dimensional approach has a number of practical and organizational advantages for service providers relative to the categorical approach as services can be arranged around limited set of key dimensions of risk that are relevant to multiple diagnostic categories (e.g., neuroticism, impulsivity, and improving response inhibition). Finally, while there is much enthusiasm for a shift to more dimensional approaches in psychopathology research and treatment, there remain a number of very important avenues of investigation that can only be addressed within the context of large and richly phenotyped and genotyped data sets, such as the question of how these dimensions interact to further influence risk for psychopathology. Despite relatively consistent findings on the bifactor model of psychopathology, more studies are needed to understand environmental and genetic contributions to these risk dimensions, and their interactions. Both twin modeling and molecular genetic studies will help to identify the biological basis of these risk dimensions, as will large neuroimaging studies.

## Supplementary Material

10.1037/abn0000193.supp

## Figures and Tables

**Table 1 tbl1:** Descriptives and Correlations Between Psychopathology Symptoms at 16 Years and Demographic Measures

Psychopathology Symptoms	1	2	3	4	5	6	7	8	9	10	11	12
1. ADHD band	1.00											
2. CD band	**.39**	1.00										
3. ODD band	**.43**	**.41**	1.00									
4. Alcohol problems	.02	**.20**	.06	1.00								
5. Number of drugs	**.13**	**.26**	.06	**.35**	1.00							
6. Smoking frequency	**.16**	**.30**	**.10**	**.37**	**.56**	1.00						
7. Gen anxiety band	**.19**	**.21**	**.10**	.07	**.15**	.12	1.00					
8. Depression band	**.30**	**.31**	**.15**	**.12**	**.22**	**.18**	**.49**	1.00				
9. Eating band	.08	**.12**	**.10**	**.09**	.08	**.09**	**.32**	**.27**	1.00			
10. OCD band	**.25**	**.21**	−.01	.05	**.13**	**.09**	**.45**	**.37**	**.23**	1.00		
11. Panic and phobias	**.24**	**.17**	**.16**	.01	.05	.05	**.44**	**.37**	**.28**	**.33**	1.00	
12. Social phobia	**.10**	**.10**	**.10**	−.03	.02	.00	**.27**	**.19**	**.18**	**.24**	**.27**	1.00
13. Gender	−.08	.00	.03	−.02	−.06	.01	**.22**	**.18**	**.31**	**.10**	**.26**	**.09**
14. Language (English vs Other)	−.05	−.05	.02	.08	−.02	−.15	−.02	−.01	**.09**	.01	−.08	**.11**
Mean	.41	1.31	.54	.62	.37	1.79	.65	.63	.58	.38	.31	1.18
*SD*	.79	.71	1.05	1.19	.77	2.31	.99	.93	.73	.66	.40	.72
Range	0–5	0–5	0–5	0–8	0–7	0–7	0–5	0–5	0–5	0–5	0–5	0–5
Likely diagnosis or case^±^, % (*n*)	3 (52)	4 (71)	7 (120)	14 (218)	26 (415)	48 (768)	8 (136)	4 (67)	1 (13)	1 (16)	1 (16)	2 (27)
*Note.* Bold indicates significant at *p* < .05. ADHD = attention-deficit/hyperactivity disorder; CD = conduct disorder; ODD = oppositional defiant disorder; Gen = general; OCD = obsessive compulsive disorder; *SD* = standard deviation. ^±^ Percentages based on observed (non missing) data at 16 years (*N* = 1,603).												

**Table 2 tbl2:** Fit Indices for Structural Equation Models of Psychopathology at 16 Years

Model	χ^2^	*df*	CFI	RMSEA	SRMR	BIC	Adj BIC
Model 1: one factor	2448.59	54	.00	.141	.080	50108.17	49901.66
Model 2a: Correlated two subfactors (EXT, INT)	1032.37	53	.59	.091	.066	49189.26	49355.97
Model 2b: Correlated three subfactors (EXT, SU, INT)	216.64	51	.93	.038	.035	48994.67	48778.62
Model 3a: Bifactor two specific (EXT, INT)	175.98	42	.94	.038	.022	48937.07	48692.43
Model 3b: Bifactor three specific (EXT, SU, INT)	277.59	42	.90	.050	.026	48957.72	48713.08
Model 3a′: Revised biactor two specific factors with correlation	170.91	41	.94	.38	.021	48938.24	48690.42
Model 3b′: Revised bifactor three specific factors with correlations	170.52	40	.94	.038	.021	48937.92	48690.10
*Note.* EXT = externalizing psychopathology; INT = internalizing psychopathology; SU = substance use. Tests of goodness of fit included the Chi-square and Comparative Fit Indices (CFI), the Standardized Root Mean Square Residual (SRMR), and the Root Mean Square Error of Approximation (RMSEA). Hu and Bentler (1999) suggest the following guidelines for interpreting Goodness-of-Fit Indices: SRMR and values close to or below .08, RMSEA values close to or below .06 and CFI close to or above .90 indicate acceptable model fit. Models were compared using the Bayesian information criterion (BIC) and the sample adjusted (adj) BIC, frequently used to compare non-nested models. Smaller values on both these measures indicate a better fitting model while penalizing for increasing model complexity.							

**Table 3 tbl3:** Standardized Factor Loadings and Significance Levels for the Bifactor Model 3a at 16 and Correlations Between Factors at 14 and 16 Years

Psychopathology symptoms	P factor at 16		EXT (SU and low ADHD) factor at 16		INT factor at 16	
	Loading	p	Loading	p	Loading	p
ADHD band	.64	.000	−.25	.008		
CD band	.65	.000	.01	.994		
ODD band	.58	.000	−.24	.073		
Drinking related problems	.26	.010	.43	.000		
Number of drugs used	.42	.000	.60	.000		
Smoking frequency	.45	.000	.60	.000		
General anxiety band	.32	.000			.60	.000
Depression band	.46	.000			.46	.000
Social phobia band	.13	.007			.34	.000
Panic and other phobias	.27	.002			.56	.000
Eating disorder band	.19	.000			.28	.000
OCD band	.29	.000			.57	.000
	*r*	*p*	*r*	*p*	*r*	*p*
Correlations between factors						
P factor at 14	.73	.000	.03	.865	−.09	.033
EXT (SU) factor at 14	.00	.988	.62	.000	.03	.660
INT factor at 14	.03	.059	.02	.543	.50	.000
*Note.* P = psychopathology; ADHD = attention-deficit/hyperactivity disorder; CD = conduct disorder; SU = substance use; EXT = externalizing psychopathology; INT = internalizing psychopathology; band: computer-generated likelihood that the individual suffers from that disorder using *DSM-IV-TR* criteria; SP = self-reported; PR = parent reported; Loading = estimated standardized factor loadings; *p* = 2-tailed significance level.						

**Table 4 tbl4:** General and Specific Associations Between Psychopathology Factors (at 16 Years) and Personality and Cognition (at 14 Years)

Personality and cognitive correlates	Unadjusted associations (bivariate correlations)							Adjusted associations (regression paths)					
	P factor		EXT (SU and low ADHD) factor		INT factor			P factor		EXT (SU and low ADHD) factor		INT factor	
	*r*	*p*	*r*	*p*	*r*	*p*		β	*p*	β	*p*	β	*p*
Personality measures at 14 years													
Anxiety sensitivity	−.03	.173	−.03	.321	**.14**	**.003**		**−.06**	**.034**	−.03	.331	**.10**	**.046**
Hopelessness	**.18**	**.000**	.03	.624	**.22**	**.000**		**.14**	**.000**	.05	.404	**.20**	**.000**
Impulsivity	**.34**	**.000**	.05	.320	.06	.057		**.31**	**.000**	.04	.602	.02	.569
Sensation-seeking	**.12**	**.007**	**.13**	**.003**	−.06	.279		.08	.064	**.14**	**.001**	−.02	.683
							*R*^*2*^	***.15***	***.000***	*.03*	*.079*	***.06***	***.009***
Novelty-seeking	**.27**	**.000**	**.26**	**.000**	**−.15**	**.000**		**.29**	**.000**	**.27**	**.000**	**−.19**	**.000**
							*R*^*2*^	***.08***	***.011***	***.07***	***.041***	***.04***	***.007***
Neuroticism	**.18**	**.000**	.03	.519	**.33**	**.000**		**.12**	**.000**	.02	.734	**.34**	**.000**
Extraversion	**.08**	**.001**	**.11**	**.000**	**−.17**	**.001**		**.19**	**.000**	**.14**	**.001**	**−.09**	**.011**
Openness	−.04	.420	.05	.269	.04	.310		.01	.816	.06	.082	.01	.815
Agreeableness	**−.27**	**.000**	−.04	.550	−.05	.290		**−.22**	**.000**	−.06	.378	.03	.499
Conscientiousness	**−.25**	**.000**	−.07	.351	.06	.001		**−.18**	**.000**	−.08	.269	**.15**	**.000**
							*R*^*2*^	***.14***	***.000***	*.03*	*.246*	***.14***	***.000***
Cognitive measures at 14 years													
Verbal IQ	**−.10**	**.014**	**.12**	**.008**	.18	.120		−.03	.484	**.13**	**.027**	.19	.170
Performance IQ	**−.14**	**.000**	.04	.232	.06	.077		**−.07**	**.025**	−.03	.540	−.03	.478
DS forward	**−.04**	**.039**	.04	.401	**.06**	**.043**		.05	.249	−.01	.955	.04	.160
DS backward	**−.10**	**.018**	.05	.117	.00	.998		−.07	.216	.03	.454	−.06	.262
Delay discounting	**.14**	**.000**	.00	.942	.01	.613		**.10**	**.015**	.02	.572	.04	.306
Risk-taking (CGT)	**.07**	**.013**	**.06**	**.045**	−.01	.843		.06	.093	**.06**	**.040**	.00	.990
RI (commission)	**.14**	**.000**	−.01	.880	−.04	.336		**.09**	**.041**	.03	.444	.00	.975
AAB (Pos-Neg Om)	−.07	.068	.03	.328	**−.10**	**.026**		−.07	.050	.04	.213	**−.09**	**.012**
Spatial WM	**.10**	**.000**	−.05	.232	−.08	.205		.02	.568	−.02	.572	−.05	.402
							*R*^*2*^	***.06***	***.003***	*.02*	*.098*	*.05*	*.427*
*Note*. P = psychopathology; EXT = externalizing; SU = substance use; INT = internalizing; IQ = intelligence quotient; DS = digit span; CGT = Cambridge Gambling Task; RI = response inhibition; AAB = affective attentional bias; Om = omission errors; WM = working memory. Bold indicates significant after controlling for multiple testing. Standardized coefficients provided: *r* = correlations and β = standardized regression paths or betas; R^2^: = variance explained in adjusted association models only (in italics). Model Fit: χ^2^(132)=643.51, CFI = .92, RMSEA = .042, SRMR = .023, for model with all personality correlates (unadjusted associations); χ^2^(78) = 423.98, CFI = .93, RMSEA = .045, SRMR = .025, for model with adjusted associations for SURPS personality traits (regression paths); χ^2^(51) = 598.23, CFI = .93, RMSEA = .070, SRMR = .027, for model with TCI novelty-seeking only (regression path); χ^2^(87) = 697.25, CFI = .92, RMSEA = .056, SRMR = .027, for model with adjusted associations for NEO-FFI personality traits (regression paths); χ^2^(124) = 350.67, CFI = .95, RMSEA = .029; SRMR = .022, for model with all cognitive measures (unadjusted associations); χ^2^(126) = 357.89, CFI = .93, RMSEA = .029; SRMR = .022, for model with all cognitive measures (adjusted associations).													

## Footnotes

1 Alternative models were also tested in which OCD and eating disorder were entered as independent variables that did not load onto the internalizing factor, but rather correlated with each other and the internalizing, externalizing and substance misuse factors (as a form of correlated model), or loading directly on the P factor and no other factors (as an alternative form of bifactor model). However, in both cases these models fit the data significantly worse than the models in which OCD and eating disorder loaded on to the internalizing factor.
