# Supplementary material for: The Structure of Psychopathology in Adolescence and Its Common Personality and Cognitive Correlates
Source: J Abnorm Psychol. 2016 Nov;125(8):1039–52. doi: 10.1037/abn0000193 (PMC5098414; doi:10.1037/abn0000193)

**Supplemental Materials**

**The Structure of Psychopathology in Adolescence and Its Common Personality and Cognitive Correlates**

**by N. Castellanos-Ryan et al., 2016, *Journal of Abnormal Psychology***

**http://dx.doi.org/10.1037/abn0000193**

**Further description of IMAGEN study**: The IMAGEN study is an ongoing European multi-center study on risk-taking behavior in teenagers. It is an integrated project funded by the European Commission in the 6th Framework Program "Life Science"(1), specifically designed to provide comprehensive behavioral, neuropsychological, genetic, functional and structural neuroimaging data related to behavioral disinhibition and reward processing in a representative sample of 2,000 young adolescents at 8 study-sites in Germany, England, France and Ireland. The recruitment strategy employed resulted in the recruitment of 40% of families who initially expressed interest in the project. Main reasons for exclusion were lack of availability of children and parents for a full assessment day at the research institute, age and contraindication for magnetic resonance imaging such as braces, premature birth, diseases of the central nervous system, brain trauma, or medication (including ADHD stimulant medication).

A total of 2,232 participants across 8 European sites were recruited via high-schools in geographical areas with minimal ethnic diversity to maximize ethnic homogeneity as a prerequisite for future genome-wide association analyses. To obtain a diverse sample in terms of socio-economic status, emotional and cognitive development, private-, state-funded schools and special educational units were equally targeted within those areas. After data quality control, complete and reliable data sets for 2,144 volunteers with an average age of 14.39 years (SD = 0.77) and an even gender ratio (*n* = 1,093 girls, i.e. 51%) were included in analyses. Table S1 gives an overview of the sample distribution across sites.

Table S1. Distribution of participants (*N*=2,144) across sites.

| Variable | |  | % | *n* |
| --- | --- | --- | --- | --- |
| Study site | England | London | 12 | 266 |
|  |  | Nottingham | 17 | 356 |
|  | Ireland | Dublin | 11 | 229 |
|  | Germany | Berlin | 12 | 263 |
|  |  | Hamburg | 12 | 255 |
|  |  | Mannheim | 12 | 262 |
|  |  | Dresden | 12 | 260 |
|  | France | Paris | 12 | 253 |

**Further description of Measures and assessment protocol:**

As the Psytools program was run at the participant’s home without direct supervision of the research team, the reliability of each individual’s data was checked in a two-stage procedure. Before every task, adolescents were asked to report on the current testing context including questions about their attentional focus and the confidentiality of the setting. Automated flags highlighted potentially problematic testing situations and were followed-up by research assistants face-to-face with the volunteer in a confidential setting. Final reliability ratings were assigned which led to in- or exclusion of the data. Exclusion criteria were: During reaction-time tasks, the child indicated that he/ she was listening to music or was exposed to disturbing noise or their mean reaction time per block within a task was below 100ms or they pressed the same response key throughout the task, during the questionnaires they indicated to have been in a hurry or somebody was watching or they indicated to have known or taken the sham drug Relevin. Also, data sets showing inconsistent responses across or extreme outliers within measures were excluded (see next section). The child self-report and the parent-report clinical screening and interview were administered under supervision at the research institutes. Participants were explained the confidential nature of the interview but answered the questions on their own to establish openness and confidentiality. Research assistants were available on demand if further support was required. Data were rated as unreliable in case the research assistant had reason to believe from their observation of the interview session that the volunteer did not answer the questions appropriately e.g. as a result of language or reading problems.

**Measures**

All measures were selected on the basis of brevity, age-appropriateness, and validity in their variant forms (English, German and French). Psychopathology and substance misuse symptoms over the last 12 months were assessed at 14 and 16 years of age. Personality and neurocognitive function were only assessed at 14 years.

**Psychopathology symptoms:** Self- and parent-report behavioral and clinical measures were assessed via online computer platforms provided by Psytools ® (Delosis Ltd, London, UK), administered at participants’ homes, and The Development and Well-Being Assessment interview (Goodman, Ford, Simmons, Gatward, & Meltzer, 2000) (see www.dawba.com), administered at the research site. As a measure of psychiatric symptoms and caseness of CD, ODD, ADHD, generalised anxiety, depression, specific phobia, social phobia, agoraphobia, panic disorder, OCD and eating disorders, the DAWBA interview was administered to adolescents and parents. Using both parent- and self-report, a prognosis for the likelihood of having a disorder was calculated by a computer algorithm. The generated band ranges from level 0 up to level 5 corresponding to the approximate prevalence rates in an epidemiological sample for the disorder in question, ranging from less than 0.1% up to 70%. Diagnostic criteria were based on the Diagnostic Statistical Manual, Version 4. The band scores of specific phobia, agoraphobia and panic disorder were averaged to create a combined “panic and other phobia” score. This was deemed justifiable as the three variables were associated with other disorders and correlates in a similar fashion at both 14 and 16 years.

**Substance misuse** was assessed using the Alcohol Use Disorders Identification Test (AUDIT; Saunders, Aasland, Babor, de la Fuente, & Grant, 1993) and the European School Survey Project on Alcohol and Drugs (ESPAD; Hibell et al, 1997). The AUDIT was developed and validated by the World Health Organization to provide a brief assessment of alcohol use disorders and was specifically designed for international use. It exists in all three languages, and has been validated on primary health care patients and community samples. For this study, the scale total for problematic or harmful alcohol use in the last year included feelings of guilt or remorse after drinking, being unable to remember what happened the night before because of drinking, being injured or having injured someone as a result of drinking and relevant others being concerned about their drinking and suggestions to cut down. The five response options range from 0 (“never”) to 4 (“daily or almost daily).

The ESPAD items used in this study comprised tobacco use frequency and the number of drugs used over the last 12 months, a composite score that included the following substances or groups of substances: marijuana or hashish, inhalants, tranquilisers or sedatives, amphetamines, LSD, magic mushrooms or hallucinogens, crack, cocaine, heroin, narcotics, ecstasy, ketamine or phenylclinidine, GHB or liquid ecstasy, and anabolic steroids.

**Personality**: Personality traits wasere assessed with the self-reported Substance Use Risk Profile Scale (SURPS; Woicik, Stewart, Pihl, & Conrod, 2009) , the NEO Five Factor Inventory (NEO-FFI; Costa Jr & McCrae, 1992) and the Temperament and Character Inventory (TCI; Cloninger, Przybeck, Svrakic, & Wetzel, 1994). The SURPS assessed the personality traits of hopelessness, anxiety sensitivity, impulsivity and sensation-seeking. The reliability and concurrent and predictive validity of this measure has now been well established in severaldifferent adolescents and adult samples in different countries (Castellanos-Ryan et al., 2013; Krank et al., 2011; Woicik et al., 2009). The NEO-FFI assessed five higher order personality characteristics of interest: neuroticism, conscientiousness, extraversion, agreeableness and openness to experience. The TCI was used to assess novelty- seeking, which is considered a good general measure of impulsive tendencies that also includes sensation-seeking tendencies (see Table S2 in supplementary material for correlations between personality and neurocognitive measures).

**IQ and neurocognitive measures**: Estimates of intelligence were derived from the vocabulary and similarities subtests (verbal IQ) and block design and matrix reasoning subtests (performance IQ) of the Wechsler Intelligence Scale for Children – Fourth Edition (WISC-IV; Wechsler, 2003). Digit Span forward and backward subtests from the WISC-IV were also administered and used to assess short-term auditory memory and attention and auditory working memory or the ability to manipulate verbal information while in temporary storage (Groth-Marnat & Baker, 2003; Reynolds, 1997).

**Poor Response Inhibition** was measured using the number of commission errors in a go-nogo Passive Avoidance Learning Paradigm (Newman & Wallace, 1993). This task asks volunteers to learn, by trial and error, to respond to “correct” numbers and to withhold a response for “wrong” numbers by rewarding correct or punishing wrong go and no-go responses. Three training blocks with numbers 1 and 2 representing the “correct” and the “wrong” number were used before each test block to demonstrate the association between their responses (hits and misses) and the outcome (winning or losing points): First, the subject was forced to respond to every trial, then to withhold the responses and finally to respond in the best possible way to gain the maximum amount of points. Following three training blocks test blocks each consisting of 10 test trials were presented, repeating the set of 8 two-digit numbers each time in a different random order. The stimuli set used for each condition was balanced across subjects. The number appeared on the screen for 3 seconds during which the subject was asked to decide whether to respond by pressing the space bar or to withhold the response. Afterwards, the number disappeared, leaving an inter-trial-interval of 1 second before the next number was displayed. At the upper left corner of the computer screen, a running score was continuously displayed. Three conditions were applied: reward only, punishment only and reward-punishment combined. Written instructions informed the subjects that the score would influence a real reward at the end of task. However, this did not apply to French families as the local ethics committee did not allow for any monetary reward, but were still motivated to win points or avoid losing points. Commission errors averaged across conditions were used as an index of response inhibition.

**Delay discounting** was assessed with the Kirby Delay Discounting Questionnaire (Kirby, Petry, & Bickel, 1999). With this measure, delay discounting is determined from 27 hypothetical choice questions for either immediate or delayed money, with the delays ranging from 7 to 186 days. Participants were instructed that, although hypothetical, to make choices as though they are actually going to receive the money they choose. The Kirby was scored as described previously by Kirby et al. (1999), with k values (an index of delay discounting) assigned according to choice patterns across the 27 items, with larger k values (geometric mean) indicating greater delay discounting of value for the delayed options.

**Spatial working memory, risky decision-making and information processing biases for positive and negative stimuli** were assessed with three tasks from the Cambridge Cognition Neuropsychological Test Automated Battery (CANTAB; Cambridge Cognition), the Spatial Working Memory (SWM), Cambridge Gambling task and affective go/ no-go task, respectively.

**SWM** is a self-ordered test that requires retention and manipulation of visuospatial information. A series of coloured boxes are presented at a random location on-screen, and may be hiding a blue chip. Participants have to find the blue chips, but they are told that once a blue chip has been uncovered under a particular coloured box, that box will never again be covering a blue chip, and thus must be avoided. In this way, the test assesses working memory by determining whether the participant remembers which boxes have already been chosen. The level of difficulty increases as the number of boxes increases. The number of between errors (i.e., choosing a box under which they have already discovered a chip) was used to assess poor spatial working memory.

In **the Cambridge Gambling Task**, participants had to guess whether a yellow token was hidden in one box within different numbers of red or blue boxes. Participants are presented with ten boxes, some red and blue, with color ratios of 9:1, 8:2, 7:3, and 6:4 varying from one trial to the next, and are asked to make a probabilistic judgment to determine the color of box under which a yellow token is hidden by placing a bet. Additionally, there are two conditions under which a participant can place a bet: ascending and descending. In the ascending condition, the amount a participant can bet increases proportionately (5%, 25%, 50%, 75%, and 95%) based on the total number of points accumulated at the time of the decision. The same holds for the descending condition, except the amount a participant can bet begins at 95% of total points accrued and proportionately decreases. A modified version of this task was used in which the time between stakes was reduced from 5 to 2 s to make the task shorter to avoid boredom effects in adolescents. The outcome measure of mean risk taking score (points) for each box ratio was used to assess risky decision-making.

Finally, **the affective go/no-go** is a task of emotional processing, in which affectively valenced words (happy and sad) are presented one at a time on screen. Participants are instructed initially to press the space bar when they see a happy word (e.g., hopeful), and after two 18-word blocks, the subject is then instructed to respond when they see a sad word (e.g., glum). The presentation of valenced words alternates every two blocks and continues for ten 18-word blocks. Performance variables-of-interest are the target (omission) errors to positive and negative words, with an attentional bias towards negative versus positive words being assessed with a difference score between omission errors to each set of stimuli. For further details on the neurocognitive tasks see http://www.cambridgecognition.com/academic/cantabsuite.

Table S2. Correlations between covariates (measured at 14 years)

|  |  | 1 | 2 | 3 | 4 | 5 | 6 | 7 | 8 | 9 | 10 | 11 | 12 | 13 | 14 | 15 | 16 | 17 | 18 |
| --- | --- | --- | --- | --- | --- | --- | --- | --- | --- | --- | --- | --- | --- | --- | --- | --- | --- | --- | --- |
| 1 | Anxiety Sens | 1.00 |  |  |  |  |  |  |  |  |  |  |  |  |  |  |  |  |  |
| 2 | Hopelessness | **0.11** | 1.00 |  |  |  |  |  |  |  |  |  |  |  |  |  |  |  |  |
| 3 | Impulsivity | 0.08 | **0.19** | 1.00 |  |  |  |  |  |  |  |  |  |  |  |  |  |  |  |
| 4 | Sensation Seek | **-0.12** | **-0.12** | **0.17** | 1.00 |  |  |  |  |  |  |  |  |  |  |  |  |  |  |
| 5 | Novelty Seek | **-0.13** | 0.03 | **0.39** | **0.24** | 1.00 |  |  |  |  |  |  |  |  |  |  |  |  |  |
| 6 | Neuroticism | **0.35** | **0.47** | **0.28** | -0.08 | -0.04 | 1.00 |  |  |  |  |  |  |  |  |  |  |  |  |
| 7 | Extraversion | -0.07 | **-0.37** | **0.11** | **0.20** | **0.35** | **-0.29** | 1.00 |  |  |  |  |  |  |  |  |  |  |  |
| 8 | Openness | 0.07 | -0.08 | **-0.11** | **0.17** | -0.05 | 0.04 | 0.02 | 1.00 |  |  |  |  |  |  |  |  |  |  |
| 9 | Agreeableness | -0.02 | **-0.29** | **-0.44** | -0.06 | **-0.17** | **-0.30** | **0.23** | **0.15** | 1.00 |  |  |  |  |  |  |  |  |  |
| 10 | Conscientious | 0.05 | **-0.39** | **-0.36** | -0.02 | **-0.41** | **-0.23** | **0.14** | **0.11** | **0.31** | 1.00 |  |  |  |  |  |  |  |  |
| 11 | Verbal IQ | -0.08 | 0.02 | **-0.09** | 0.04 | -0.01 | -0.07 | -0.06 | **0.31** | 0.08 | -0.02 | 1.00 |  |  |  |  |  |  |  |
| 12 | Performance IQ | -0.02 | 0.00 | -0.09 | 0.03 | -0.05 | -0.05 | -0.06 | **0.21** | 0.07 | 0.00 | **0.46** | 1.00 |  |  |  |  |  |  |
| 13 | DS forward | -0.04 | 0.00 | -0.02 | 0.02 | 0.04 | -0.02 | 0.03 | **0.11** | 0.02 | -0.03 | **0.23** | **0.23** | 1.00 |  |  |  |  |  |
| 14 | DS back | -0.03 | -0.03 | -0.08 | -0.03 | -0.02 | -0.04 | 0.02 | 0.07 | 0.06 | 0.06 | **0.22** | **0.31** | **0.50** | 1.00 |  |  |  |  |
| 15 | DD (K mean) | -0.01 | 0.01 | **0.09** | -0.01 | **0.09** | 0.02 | -0.01 | **-0.14** | **-0.12** | -0.04 | **-0.14** | **-0.15** | **-0.09** | -0.08 | 1.00 |  |  |  |
| 16 | Risk-taking | -0.04 | -0.02 | **0.10** | **0.10** | 0.08 | **-0.10** | **0.10** | **-0.12** | -0.07 | 0.01 | -0.05 | -0.08 | -0.03 | -0.02 | 0.05 | 1.00 |  |  |
| 17 | Spatial WM | 0.06 | 0.03 | **0.09** | -0.04 | 0.03 | **0.09** | 0.02 | **-0.10** | -0.08 | 0.00 | **-0.29** | **-0.40** | **-0.21** | **-0.21** | **0.13** | 0.06 | 1.00 |  |
| 18 | RI (commision) | 0.07 | -0.02 | **0.11** | -0.05 | 0.04 | **0.09** | 0.02 | -0.07 | **-0.09** | -0.04 | **-0.22** | **-0.28** | **-0.23** | **-0.22** | **0.17** | 0.01 | **0.29** | 1.00 |
| 19 | AAB (P-N Om) | -0.03 | -0.04 | -0.04 | -0.03 | -0.02 | -0.03 | -0.04 | -0.05 | 0.02 | 0.01 | -0.04 | -0.07 | -0.03 | 0.00 | -0.02 | 0.00 | 0.05 | 0.02 |

Bold indicates significant at p>.05; IQ: Intelligence Quotient; DS: digit span; CGT: Cambridge Gambling Task; RI: Response Inhibition; AAB: Affective Attentional bias; P-N Om: Positive minus Negative Omission errors; WM: Working Memory.

Table S3. Standardized factor loadings and significance levels for the modified bi-factor model with three specific factors and covariance between specific factors (model 3b’) at age 16

|  | P factor at 16 | | EXT factor at 16 | | | SU factor at 16 | | | INT factor at 16 | | |
| --- | --- | --- | --- | --- | --- | --- | --- | --- | --- | --- | --- |
|  | Loading | p | | Loading | P | | Loading | P | | Loading | P |
| ADHD band | 0.58 | 0.000 | | 0.36 | 0.000 | |  |  | |  |  |
| CD band | 0.62 | 0.000 | | 0.14 | 0.018 | |  |  | |  |  |
| ODD band | 0.53 | 0.000 | | 0.35 | 0.002 | |  |  | |  |  |
| Drinking related problems | 0.37 | 0.007 | |  |  | | 0.35 | 0.001 | |  |  |
| Number of drugs used | 0.57 | 0.000 | |  |  | | 0.45 | 0.016 | |  |  |
| Smoking frequency | 0.60 | 0.000 | |  |  | | 0.46 | 0.022 | |  |  |
| General Anxiety band | 0.37 | 0.000 | |  |  | |  |  | | 0.57 | 0.000 |
| Depression band | 0.48 | 0.000 | |  |  | |  |  | | 0.44 | 0.000 |
| Social Phobia band | 0.15 | 0.001 | |  |  | |  |  | | 0.34 | 0.000 |
| Panic and other phobias | 0.31 | 0.000 | |  |  | |  |  | | 0.55 | 0.000 |
| Eating Disorder band | 0.22 | 0.000 | |  |  | |  |  | | 0.26 | 0.000 |
| OCD band | 0.32 | 0.000 | |  |  | |  |  | | 0.55 | 0.000 |
|  |  |  | |  |  | |  |  | |  |  |

Legend: P – psychopathology; ADHD – Attention Deficit Hyperactivity Disorder; CD – Conduct Disorder; SM – Substance Misuse; EXT - externalizing psychopathology; INT – internalizing psychopathology; band: computer-generated likelihood that the individual suffers from that disorder using DSM-IV-TR criteria; SP – self reported; PR – parent reported; Loading – estimated standardized factor loadings; p – 2-tailed significance level.

Table S4. Standardized factor loadings and significance levels for the bi-factor with two specific factors and no covariation between specific factors (model 3a) at 14 years

|  | P factor at 14 | | EXT (SU and low ADHD/ODD) factor at 14 | | INT factor at 14 | |
| --- | --- | --- | --- | --- | --- | --- |
|  | Loading | p | Loading | P | Loading | P |
| ADHD band | 0.64 | 0.000 | -0.32 | 0.001 |  |  |
| CD band | 0.65 | 0.000 | 0.11 | 0.194 |  |  |
| ODD band | 0.68 | 0.000 | -0.31 | 0.001 |  |  |
| Drinking related problems | 0.27 | 0.000 | 0.38 | 0.000 |  |  |
| Number of drugs used | 0.31 | 0.000 | 0.43 | 0.000 |  |  |
| Smoking frequency | 0.50 | 0.000 | 0.55 | 0.000 |  |  |
| General Anxiety band | 0.22 | 0.000 |  |  | 0.66 | 0.000 |
| Depression band | 0.27 | 0.000 |  |  | 0.45 | 0.000 |
| Social Phobia band | 0.07 | 0.001 |  |  | 0.36 | 0.000 |
| Panic and other phobias | 0.13 | 0.000 |  |  | 0.35 | 0.000 |
| Eating Disorder band | 0.16 | 0.000 |  |  | 0.29 | 0.000 |
| OCD band | 0.15 | 0.000 |  |  | 0.48 | 0.000 |

Legend: P – psychopathology; ADHD – Attention Deficit Hyperactivity Disorder; CD – Conduct Disorder; SM – Substance Misuse; INT – internalizing psychopathology; band: computer-generated likelihood that the individual suffers from that disorder using DSM-IV-TR criteria; Loading – estimated standardized factor loadings; p – 2-tailed significance level.

Table S5. Associations between psychopathology factors (at 16 years) and personality and cognition (at 14 years) in a bifactor model with three specific factors and covariance between specific factors (model 3b’)

|  | P factor | | | | | EXT factor | | | | | | | SU factor | | | | | | INT factor | | | | |  |
| --- | --- | --- | --- | --- | --- | --- | --- | --- | --- | --- | --- | --- | --- | --- | --- | --- | --- | --- | --- | --- | --- | --- | --- | --- |
|  | r | | | p | | | r | | | P | | | | r | | | p | | | r | | | p | |
| Personality Measures at 14 years: | | | | |  | | | | | | |  | | | | | |  | | | | | |  |
| Anxiety Sensitivity | -0.03 | | 0.245 | | | -0.01 | | | 0.906 | | | | **-0.04** | | **0.005** | | | | **0.15** | | | **0.002** | |  |
| Hopelessness | **0.21** | | **0.000** | | | 0.01 | | | 0.897 | | | | 0.03 | | 0.652 | | | | **0.19** | | | **0.000** | |  |
| Impulsivity | **0.30** | | **0.000** | | | **0.16** | | | **0.017** | | | | 0.10 | | 0.060 | | | | 0.03 | | | 0.839 | |  |
| Sensation-seeking | 0.11 | | 0.121 | | | -0.07 | | | 0.267 | | | | **0.11** | | **0.018** | | | | -0.12 | | | 0.122 | |  |
|  |  | |  | | |  | | |  | | | |  | |  | | | |  | | |  | |  |
| Novelty-seeking | 0.21 | | 0.087 | | | 0.06 | | | 0.575 | | | | **0.33** | | **0.000** | | | | **-0.23** | | | **0.000** | |  |
|  |  | |  | | |  | | |  | | | |  | |  | | | |  | | |  | |  |
| Neuroticism | **0.12** | | **0.008** | | | 0.03 | | | 0.405 | | | | 0.04 | | 0.281 | | | | **0.37** | | | **0.000** | |  |
| Extraversion | **0.17** | | **0.003** | | | 0.02 | | | 0.696 | | | | **0.18** | | **0.000** | | | | **-0.13** | | | **0.000** | |  |
| Openness | 0.00 | | 0.993 | | | -0.01 | | | 0.862 | | | | 0.05 | | 0.236 | | | | 0.02 | | | 0.682 | |  |
| Agreeableness | -0.14 | | 0.053 | | | **-0.14** | | | **0.006** | | | | -0.12 | | 0.052 | | | | 0.02 | | | 0.621 | |  |
| Conscientiousness | **-0.15** | | **0.019** | | | -0.06 | | | 0.230 | | | | **-0.13** | | **0.030** | | | | **0.18** | | | **0.000** | |  |
|  |  | |  | | |  | | |  | | | |  | |  | | | |  | | |  | |  |
| Cognitive Measures at 14 years: | |  | | | | | |  | | |  | | | | |  | | | | |  | | |  |
| Verbal IQ | -0.10 | | 0.115 | | | 0.06 | | | 0.669 | | | | **0.17** | | **0.002** | | | | 0.22 | | | 0.103 | |  |
| Performance IQ | -0.09 | | 0.058 | | | -0.06 | | | 0.616 | | | | -0.04 | | 0.455 | | | | -0.04 | | | 0.527 | |  |
| DS forward | -0.03 | | 0.584 | | | 0.15 | | | 0.093 | | | | 0.02 | | 0.560 | | | | **0.12** | | | **0.021** | |  |
| DS backward | -0.12 | | 0.182 | | | 0.01 | | | 0.924 | | | | 0.05 | | 0.195 | | | | -0.03 | | | 0.455 | |  |
| Delay discounting | **0.14** | | **0.005** | | | -0.03 | | | 0.877 | | | | 0.03 | | 0.925 | | | | 0.01 | | | 0.750 | |  |
| Risky-taking (CGT) | 0.03 | | 0.606 | | | 0.08 | | | 0.469 | | | | **0.08** | | **0.014** | | | | 0.02 | | | 0.787 | |  |
| RI (commission) | **0.15** | | **0.000** | | | 0.04 | | | 0.651 | | | | -0.01 | | 0.839 | | | | -0.04 | | | 0.137 | |  |
| AAB (Pos-Neg Om) | -0.07 | | 0.078 | | | -0.03 | | | 0.662 | | | | 0.04 | | 0.193 | | | | **-0.10** | | | **0.031** | |  |
| Spatial WM | **0.13** | | **0.017** | | | -0.07 | | | 0.360 | | | | -0.07 | | 0.250 | | | | -0.10 | | | 0.108 | |  |

P: Psychopathology; EXT: externalizing; INT: internalizing; IQ: Intelligence Quotient; DS: digit span; CGT: Cambridge Gambling Task; RI: Response Inhibition; AAB: Affective Attentional bias; Om: Omission errors; WM: Working Memory. Standardized coefficients provided: r: correlation.

Figure S1. One general psychopathology factor model (model 1).


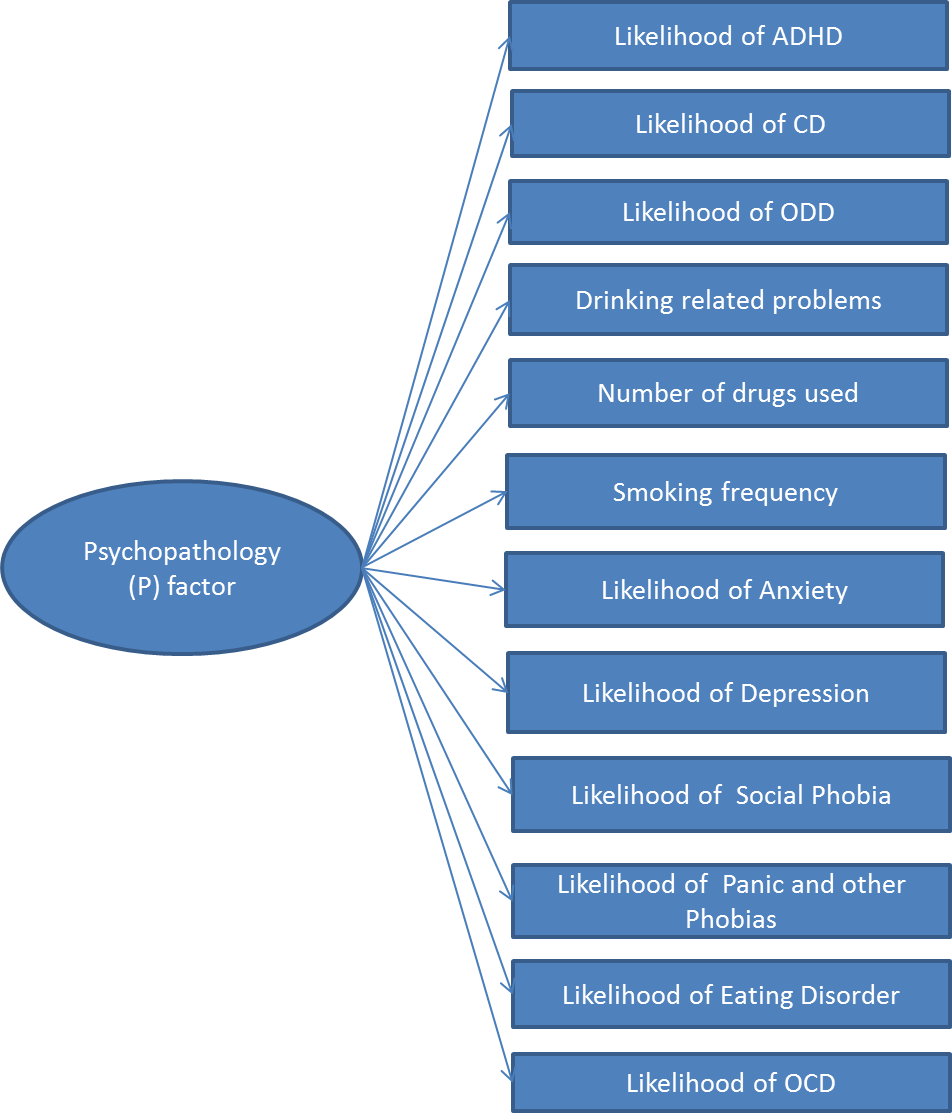


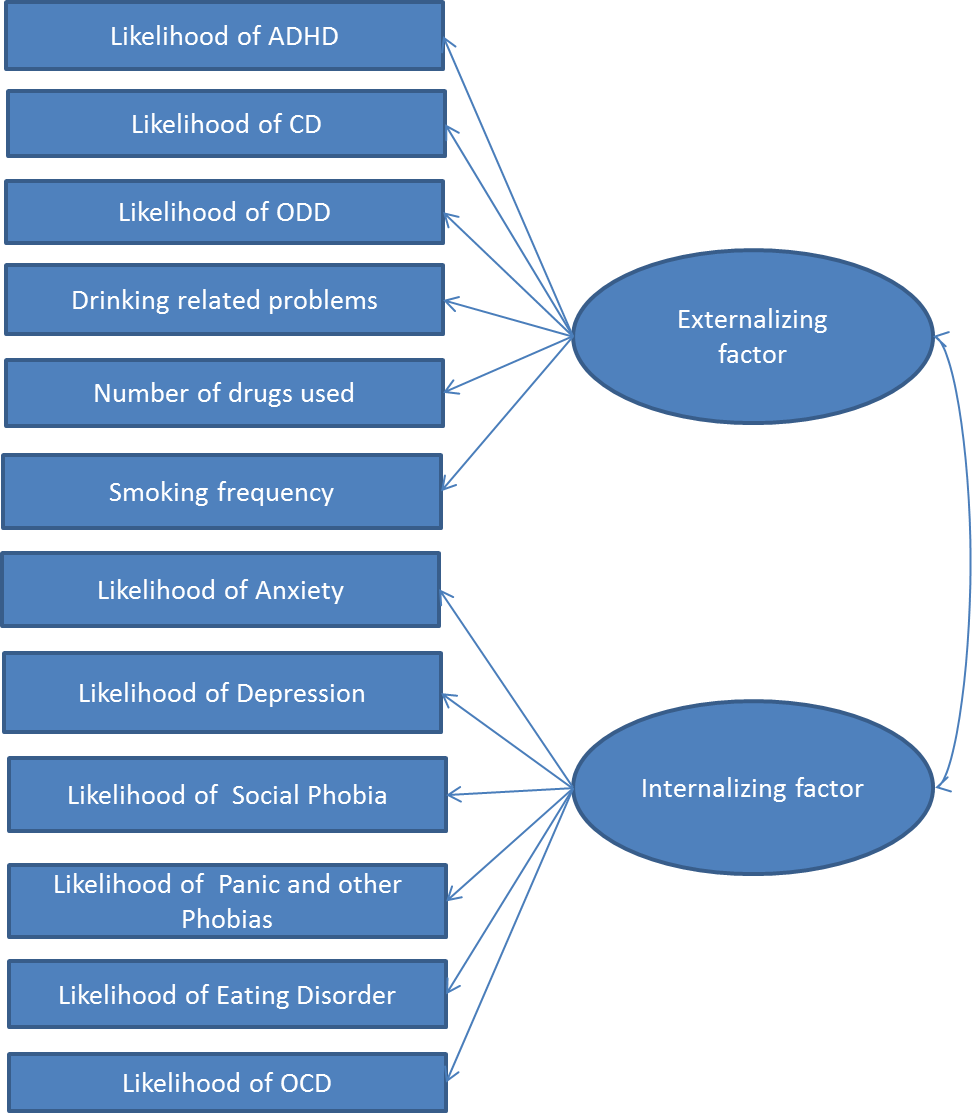
Figure S2. Two correlated factors model of psychopathology (model 2a).

Figure S3. Three correlated factors model of psychopathology (model 2b).


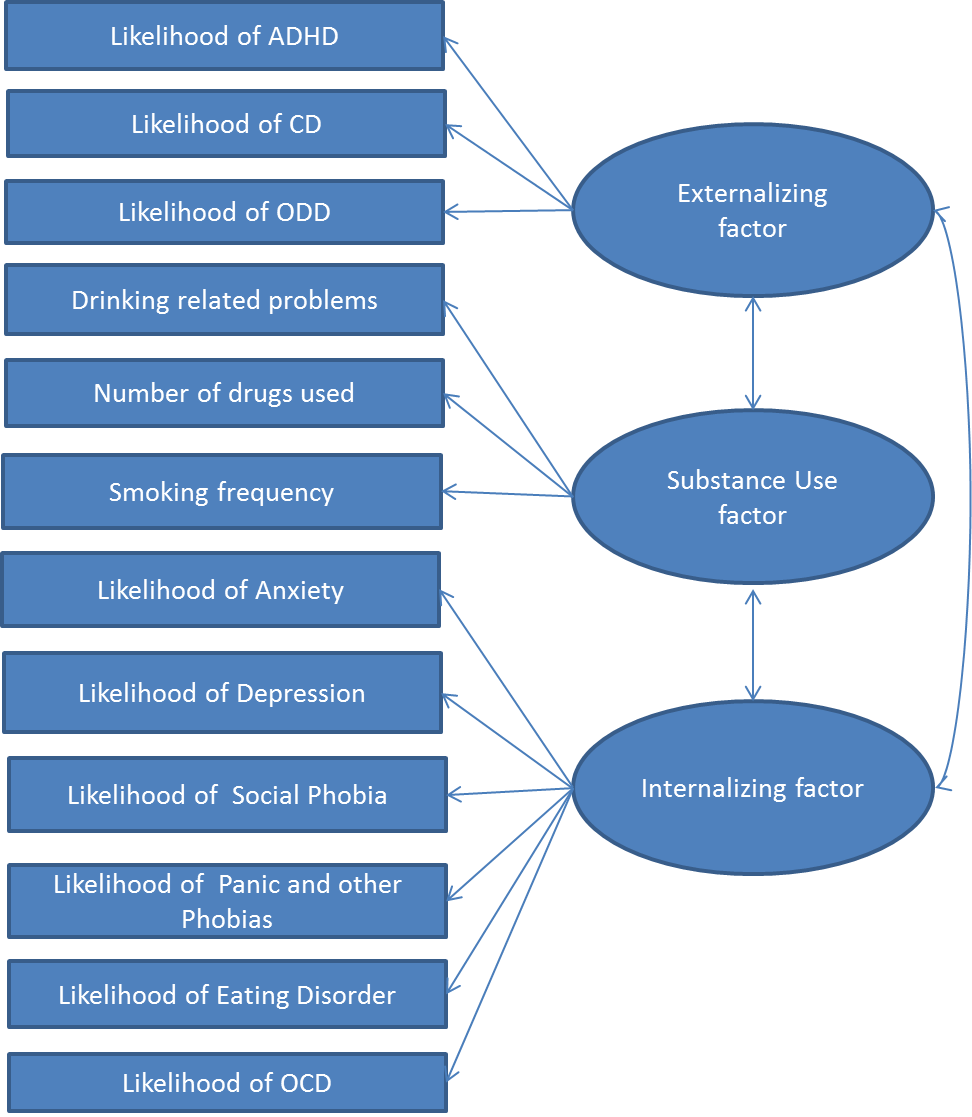


Figure S4. Bi-factor two specific factors model of psychopathology (model 3a)


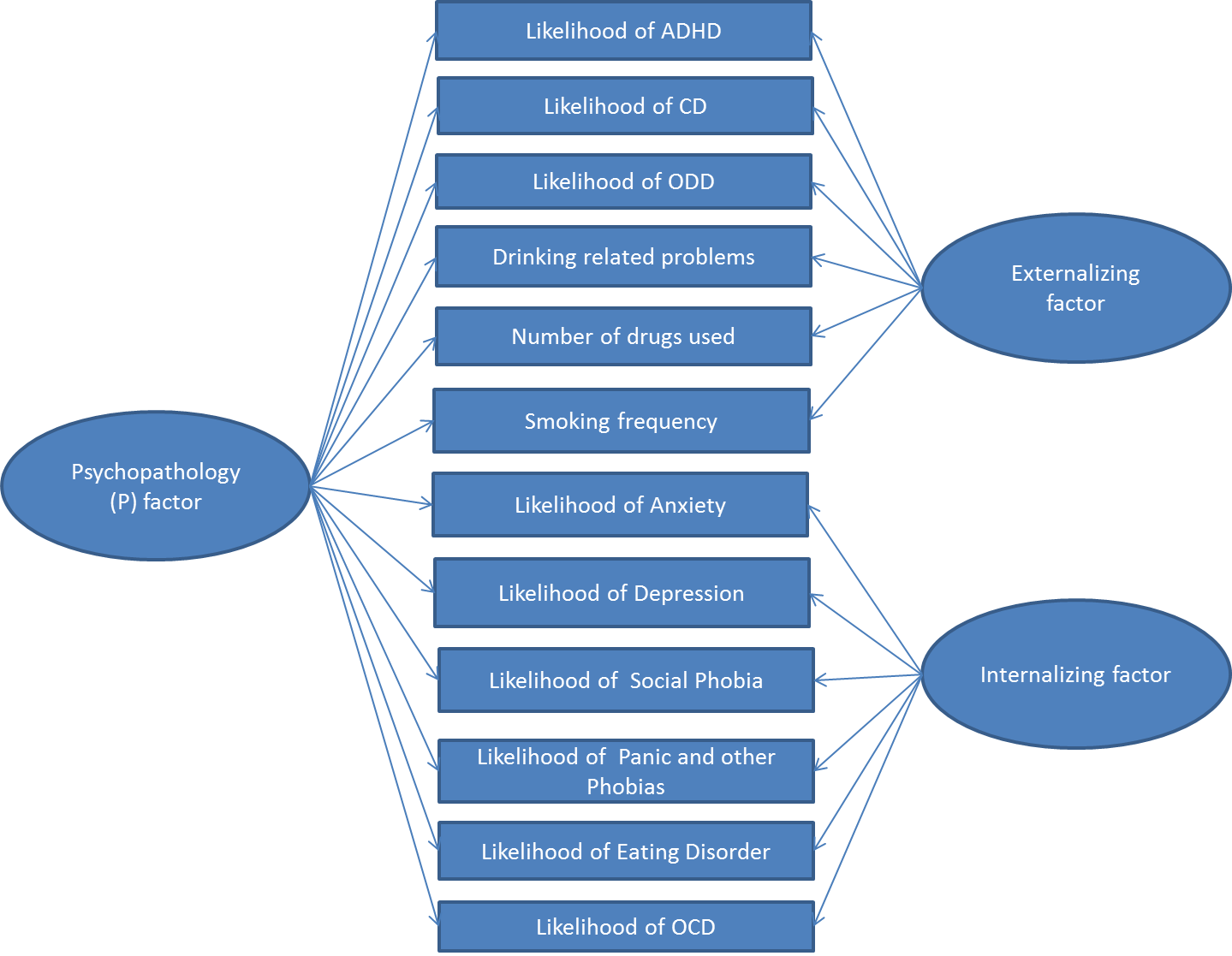


Figure S5. Bi-factor three specific factors model of psychopathology (model 3b)


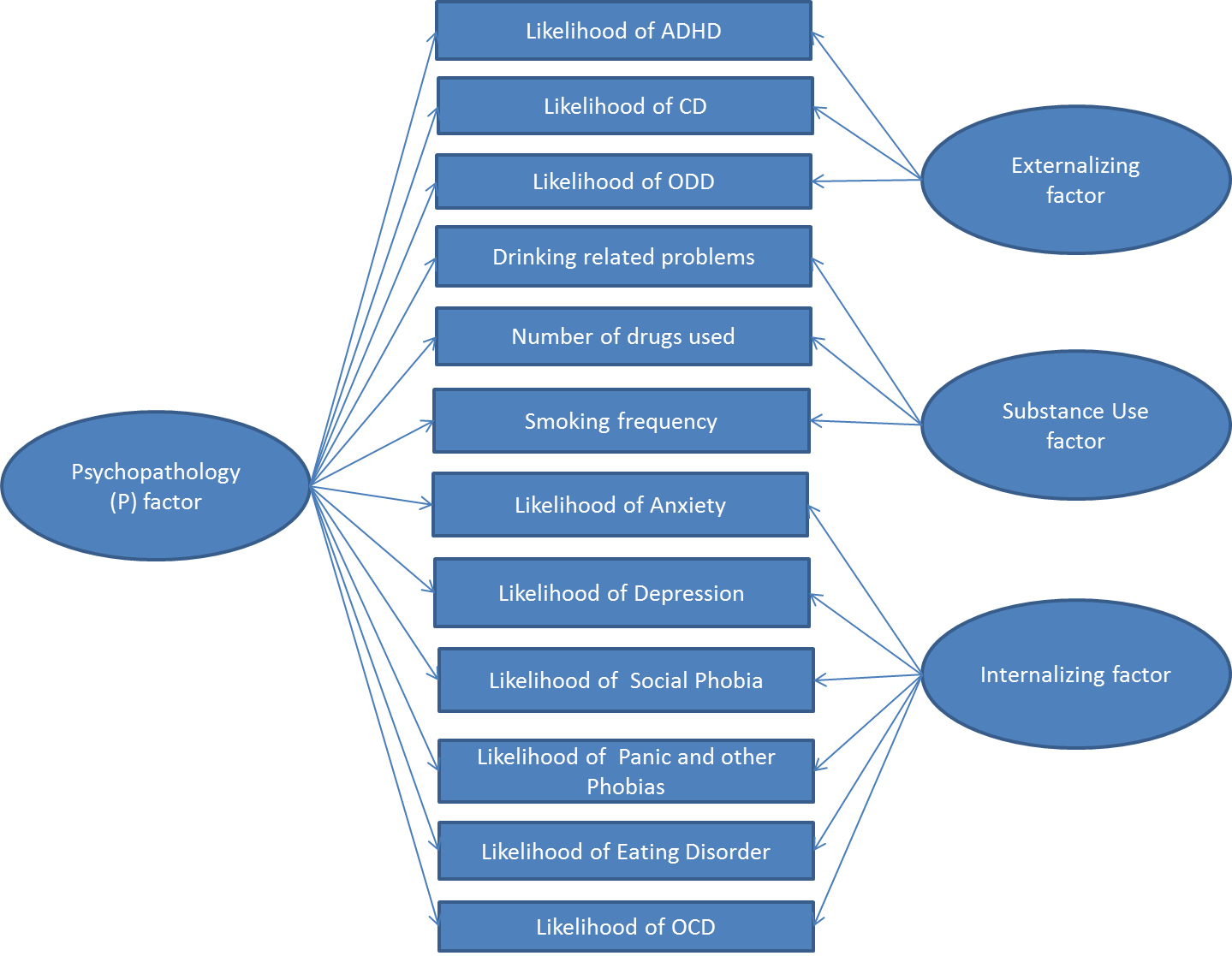


Figure S6. Modified bi-factor two specific factors model of psychopathology (model 3a’)


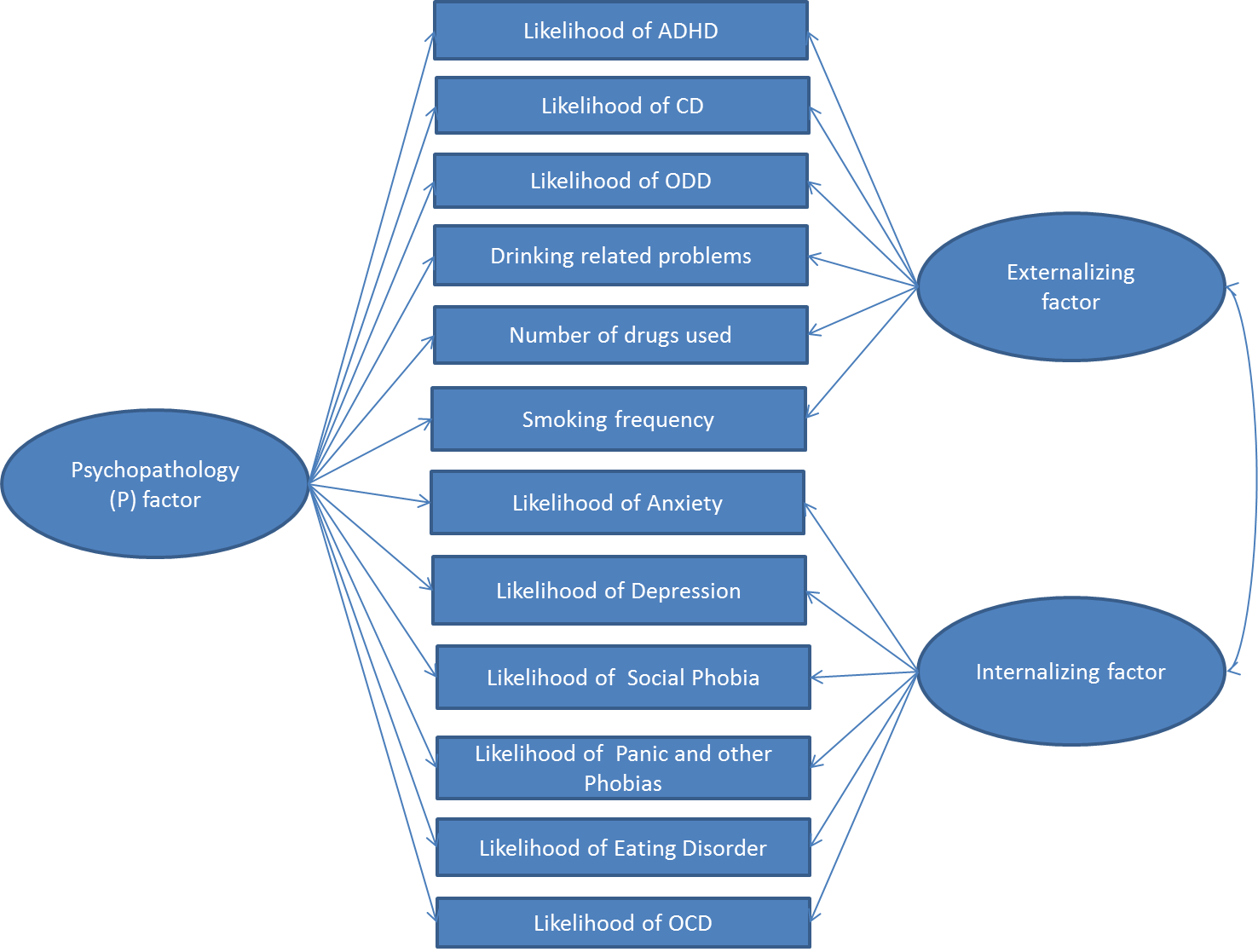


Figure S7. Modified bi-factor three specific factors model of psychopathology (model 3b’)


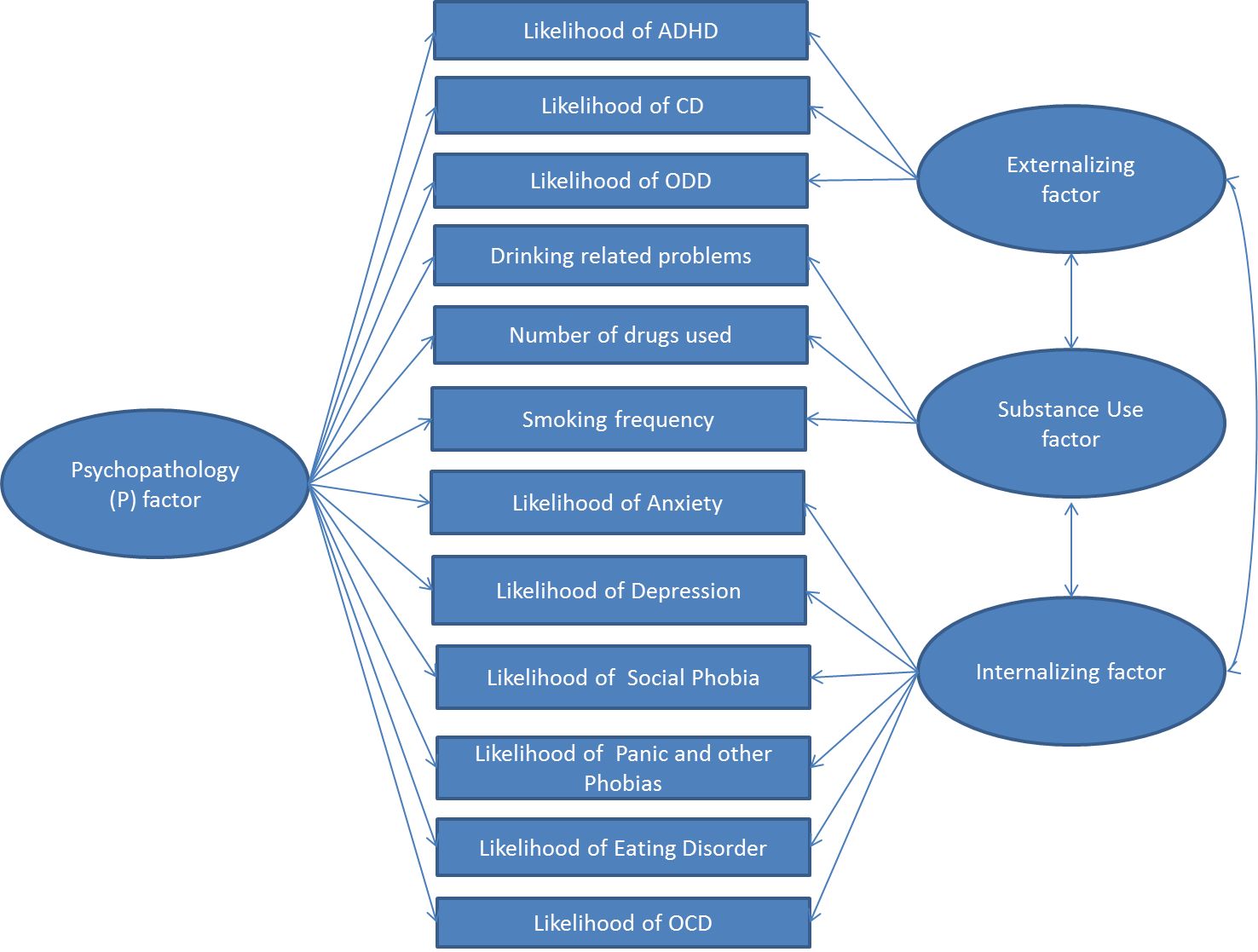

Supplement: Supplementary file 1 [file z2l006162977so1.docx]
